# Proteolytic EphA2 fragments cooperatively promote hepatocellular carcinoma progression

**DOI:** 10.1038/s41419-026-09024-1

**Published:** 2026-06-29

**Authors:** Kazuki Ikeda, Nobuhiko Asakura, Soyogi Sengoku, Eiki Tsukamoto, Nobuaki Funahashi, Naohiko Koshikawa

**Affiliations:** 1 School of Life Science and Technology, Institute of Science Tokyo, Yokohama, Japan; 2 Center for Mathematical Modeling and Data Science, The University of Osaka, Toyonaka, Japan; 3 Kanagawa Cancer Center Research Institute, Yokohama, Japan

**Keywords:** Liver cancer, Liver cancer

## Abstract

EphA2 is a receptor tyrosine kinase that suppresses tumor growth when bound by its ligand ephrin-A1 (EA1), but promotes tumor progression in the absence of ligand. In hepatocellular carcinoma (HCC) cells, EphA2 is proteolytically cleaved by membrane-type 1 matrix metalloproteinase (MT1-MMP), producing a C-terminal fragment (EphA2-CF) and an N-terminal fragment (EphA2-NF). The functional role of these cleavage fragments on HCC remains unclear. Herein, we investigated their roles in hepatocarcinogenesis and malignant progression. Western blotting and membrane biotinylation assays revealed that EphA2-CF was present in HCC cells co-expressing EphA2 and MT1-MMP. EphA2-CF-expressing cells were resistant to EA1-induced growth suppression, while MT1-MMP knockdown restored EA1 sensitivity. In Hep3B cells, stable EphA2-CF expression confirmed resistance to EA1-mediated inhibition of proliferation and survival. Mechanistically, EphA2-CF sustained oncogenic signaling through constitutive phosphorylation at EphA2-S^897^, while failing to induce Y^588^ phosphorylation after EA1 stimulation. Mutation of EphA2-S^897^ abolished EphA2-CF-driven proliferation and survival. Reverse-phase protein array identified activation of the EGFR-AKT axis and inactivation of GSK3β as key downstream events. Both pharmacological inhibition of EGFR or AKT, and activation of GSK3β suppressed EphA2-CF-driven proliferation. Furthermore, enforced expression of a constitutively active GSK3β mutant (S9A) markedly suppressed EphA2-CF–driven tumorigenesis in mice, genetically validating GSK3β inactivation as a critical effector of EphA2-CF oncogenic signaling. In addition, soluble EphA2-NF functioned as a decoy receptor for EA1, blocking its tumor-suppressive activity. These findings demonstrate that MT1-MMP–mediated EphA2 processing promotes HCC malignancy via dual mechanisms: EphA2-CF drives ligand-independent EGFR/AKT/GSK3β signaling, while EphA2-NF inhibits EA1-mediated tumor suppression.

## Introduction

EphA2 mediates opposing intracellular signaling, promoting cancer progression in the absence of ephrin-A ligands while suppressing tumor growth when ligands are present [1, 2]. Its expression is markedly elevated in cancer cells and related tissues compared with normal epithelial cells, correlating with malignancy [3]. Consequently, EphA2 has become an attractive therapeutic target for molecular cancer, leading to the development of multiple EphA2-targeted drugs [4, 5]. However, none have demonstrated sufficient clinical efficacy [3, 6].

This limited efficacy is generally attributed to the complex dual functions of EphA2, which can suppress or promote cancer depending on ligand availability. Recent studies have reported the presence of soluble ephrin-A proteins in the serum of cancer patients and within the tumor microenvironment, where they induce ligand-dependent, tumor-suppressive EphA2 signaling [7–10]. However, the mechanisms by which cancer cells selectively regulate ligand-dependent versus ligand-independent EphA2 signaling remain largely unknown. MT1-MMP is a metalloprotease present on the membrane of cancer cells, whose expression increases with malignancy [11, 12]. It is a key mediator of cancer invasion and metastasis and is highly localized at the invasive front of malignant tumors [11, 13]. Although increasing evidence suggests that MT1-MMP also regulates proliferation, survival, and chemoresistance, through remodeling of the extracellular matrix and processing of membrane proteins [14]. These findings highlight its crucial role in cancer progression.

Sugiyama et al. demonstrated that MT1-MMP cleaves EphA2 in breast cancer cells, thereby enhancing cell motility by activating the Src and RhoA signaling pathways [15]. Our study also demonstrated that EphA2 interacts with MT1-MMP on the surface of cancer cells, where MT1-MMP cleaves the N-terminal ligand-binding domain of EphA2 [16, 17]. This cleavage produces a truncated C-terminal EphA2 fragment that activates ligand-independent cancer-promoting signals. Notably, inhibition of MT1-MMP-mediated EphA2 cleavage significantly reduced cancer cell proliferation and lung metastasis in a mouse model [16, 17].

In addition to MT1-MMP, ADAM-9 (a disintegrin and metalloproteinase), ADAM-12, ADAM-17, and the fibrinolytic enzyme coagulation factor VIIa/tissue factor complex have been implicated in the regulation of EphA2 via the cleavage of ephrin A1 (EA1) or EphA2 in glioblastoma, lung, and colorectal cancers [18–21]. These findings suggest that the proteolytic processing of EphA2 plays a pivotal role in the development of malignancy.

EphA2-NF and EphA2-CF, generated through the interaction between MT1-MMP and EphA2, represent cancer-specific post-translational modifications and can serve as biomarkers for cancer detection. Our previous study indicated that serum EphA2-NF levels could be a potential biomarker for early-stage pancreatic cancer [22]. Furthermore, immunohistochemical analysis of surgically resected precancerous intraductal papillary mucinous neoplasms (IPMNs) revealed loss of the EphA2 N-terminal domain in lesions containing adenocarcinoma, while EphA2 cleavage was not observed in non-malignant IPMNs [22]. This suggests that EphA2 processing may contribute not only to malignancy progression but also to carcinogenesis. However, a systematic analysis of how EphA2 cleavage fragments influence the transition from carcinogenesis to malignancy is lacking. In this present study, we investigated the molecular mechanisms and signaling pathways by which two proteolytic fragments, EphA2-NF and EphA2-CF, regulate oncogenesis and the acquisition of malignant traits in hepatocellular carcinoma (HCC) cells. Given that EphA2 is highly expressed in HCC and plays a key role in malignant transformation, HCC cells provide a suitable model to investigate these processes [23, 24].

## Methods

### Cell lines and culture conditions

Human hepatocellular carcinoma (HCC) cell lines (JHH2, JHH4, JHH5, JHH6, JHH7, and Huh1) were obtained from the JCRB Cell Bank (National Institutes of Biomedical Innovation, Health and Nutrition, Osaka, Japan). Hep3B and SK-Hep-1 cell lines were purchased from the American Type Culture Collection (ATCC, Manassas, VA, USA). JHH2, JHH4, JHH7, Huh1, SK-Hep-1, and Hep3B cells were cultured in low-glucose Dulbecco’s modified Eagle’s medium (DMEM) supplemented with 10% fetal bovine serum (FBS) and 1% penicillin-streptomycin. JHH5 and JHH6 cells were cultured in William’s E medium (Thermo Fisher Scientific) supplemented with 10% FBS and 1% penicillin-streptomycin. All cells were maintained in 5% CO_2_ at 37 °C and were tested for mycoplasma contamination via PCR.

### Cell growth assays

Two-dimensional (2-D) cell growth assays were performed as described previously [16, 25, 26]. Cell growth was assessed both as a time-course and at the endpoint. Hep3B, JHH4, and SK-Hep1 cells were seeded at 2 × 10⁴, 3 × 10⁴, and 1 × 10⁴ cells/well, respectively, in a 24-well plate and cultured for 3, 5, or 7 days. For endpoint analyses, 3 × 10⁴ cells/well were seeded and counted after 5 days. At each time point, viable cells were quantified by trypan blue staining and microscopy.

For three-dimensional (3-D) cultures, collagen gels were prepared with collagen type I, 10× MEM, and neutralization buffer, then added to 24-well plates, and polymerized at 37 °C. Next, 1 × 10^5^ Hep3B cells were added to the gel and further polymerized at 37 °C. Finally, the cells were incubated in growth medium containing 10% FBS. After incubation for 10 days, the collagen gel was dissolved in 1% type I collagenase (Gibco Life Technologies, Grand Island, NY, USA) in PBS, and viable cells were counted using trypan blue staining and microscopy.

For the spheroid growth assay, poly-(2-hydroxyethyl methacrylate) (poly-HEMA)-coated 96-well round-bottom plates were prepared using 30 mg/mL poly-HEMA dissolved in 95% ethanol. Hep3B cells were added at a density of 1 × 10^3^ cells/well and incubated in a growth medium containing 10% FBS for 12 days. Spheroid diameters were measured, and growth activity was determined using the formula: V = 4/3πr^3^ (where V is tumor volume, and r is tumor radius).

### Plasmids

The coding sequences of human EphA2, EphA2-NF and EphA2-CF were cloned into the pENTR-TOPO vector with a C-terminal FLAG- or HA-tag. The EphA2-CF-S897A inactive mutant was introduced into pENTR-EphA2-CF-HA via PCR. The constitutively active GSK3β-S9A mutant was cloned into the pENTR-TOPO vector with a C-terminal Myc-tag. The coding sequence of human Ephrin-A1 was cloned into the pENTR-TOPO vector with a C-terminal Fc-tag. Entry clones for each gene were integrated into pLenti6-DEST using Gateway cloning technology (Thermo Fisher Scientific).

### Gene knockdown and overexpression

Short hairpin RNA (shRNA) constructs targeting MT1-MMP were purchased from Sigma-Aldrich. The Vira Power Lentiviral Expression System (Thermo Fisher Scientific) was used to stably knock down the MT1-MMP via shRNA in SK-Hep1 cells, and the cells were maintained in 1 µg/mL Puromycin (Sigma-Aldrich). Two shRNA sequences were used: 5’- GTGGTGTTCCAGACAAGTTTG -3’ (pLKO1-puro-shMT1-MMP#1) and 5’-CATTGCATCTTCCCTAGATAG -3’ (pLKO1-puro-shMT1-MMP#2).

For overexpression, Hep3B cells stably expressing EphA2, EphA2-CF, and EphA2-CF-S897A were generated using the Vira Power Lentiviral Expression System (Thermo Fisher Scientific) according to the manufacturer’s instructions. Similarly, SK-Hep1 cells stably expressing the activated GSK3β mutant (S9A) were generated. Hep3B cells transiently expressing GSK3β-S9A were introduced using Lipofectamine 3000 (Invitrogen) according to the manufacturer’s instructions.

### Xenografts

Cell tumorigenicity was examined in 6-week-old male BALB/c nude mice. Briefly, 1 × 10^7^ transfectant cells in the exponential growth phase were suspended in 100 µL of growth medium and injected subcutaneously into the mice (6 mice/group, 2 spots/mouse). The implanted tumor volume was measured weekly using a caliper, and the following formula was applied: V = (LW^2^) π/6, where V, volume (mm^3^); L, biggest diameter (mm); W, smallest diameter (mm). The mice were euthanized at 5-weeks post-tumor inoculation.

### Statistical analysis

All data are presented as means ± SD or SEM from at least three independent experiments. Statistical significance was assessed using unpaired Student’s *t*-tests or Tukey-Kramer multiple-comparison tests, as appropriate. Analyses were performed using IBM SPSS Statistics version 29.0.0.0 (IBM, Armonk, NY, USA), and *P* values were calculated.

## Results

### Expression of wild-type EphA2, EphA2-CF and MT1-MMP in HCC cells

**Fig. 1:**
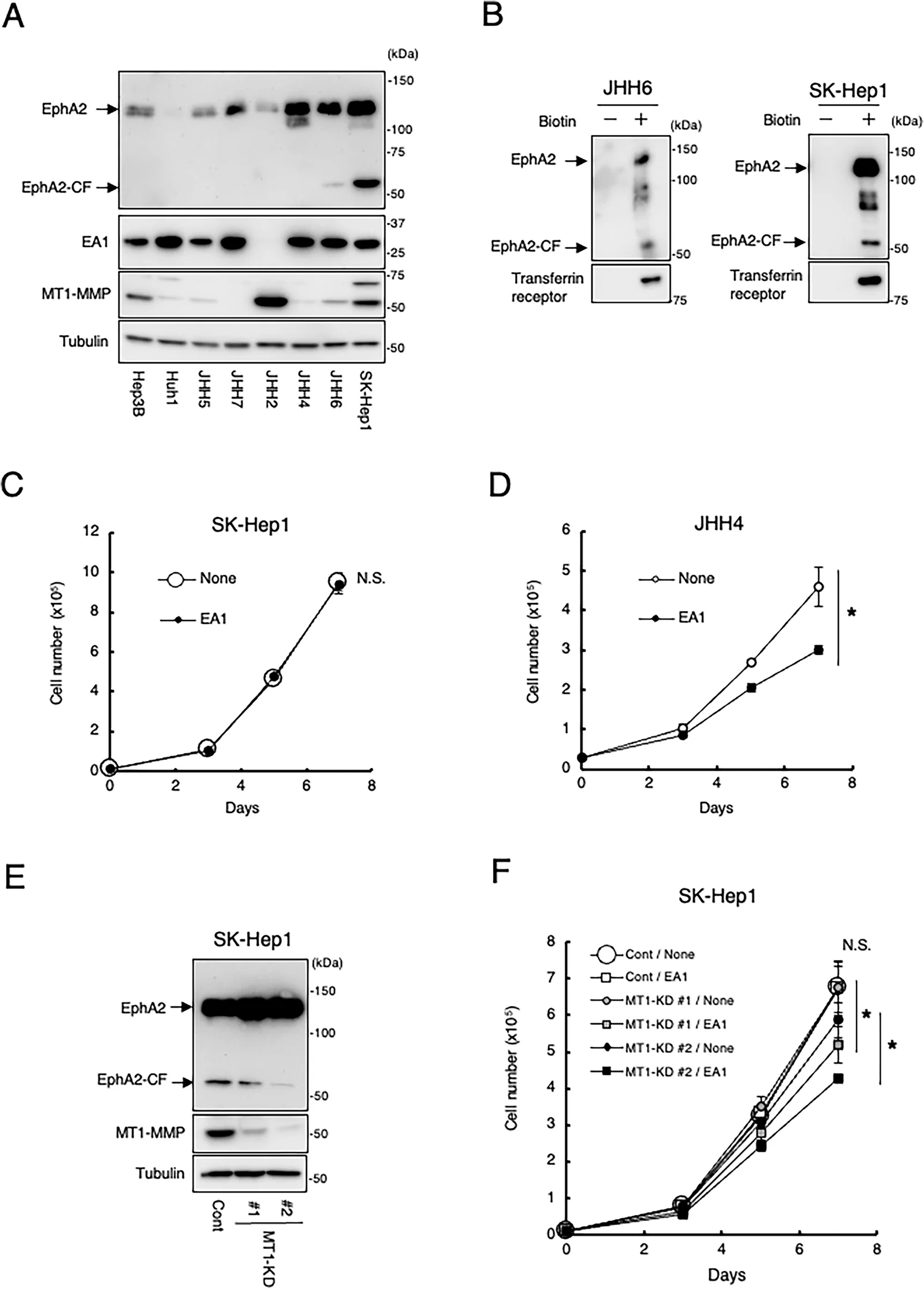
MT1-MMP and EphA2 are resistant to EA1-mediated growth suppression in HCC. **A** Detection of EphA2, EphA2-CF, MT1-MMP, and EA1 in eight HCC cell lines. β-tubulin was used as an internal control. **B** Expression analysis of EphA2 and EphA2-CF in membrane protein extracts from JHH6 and SK-Hep1 cell lines. The transferrin receptor was used as a positive control. **C, D** Comparison of cell growth of SK-Hep1 and JHH4 cell lines cultured in 24-well plates in the presence or absence of EA1. Cell counts were performed after incubation for 3, 5, and 7 days. **E** Cell lysates from MT1-MMP knockdown (MT1-KD) and control shRNA (Cont) SK-Hep1 cells were immunoblotted for MT1-MMP and EphA2. β-tubulin was used as an internal control. **F** Proliferation analysis of the cells described in (**E**) in the presence or absence of EA1. Cell counts were performed after incubation for 3, 5, and 7 days. All experiments were performed in duplicate, and at least three independent experiments were conducted. Data are presented as mean ± SD (**C, D, F**). Statistical analysis was performed using Student’s *t*-test or one-way ANOVA with Tukey’s multiple comparisons test (*p < 0.05).

We first examined the expression of wild-type EphA2 (EphA2), EphA2-CF, MT1-MMP, and ephrin-A1 (EA1) via western blotting (WB) with an EphA2 monoclonal antibody (mAb) (C3) in eight HCC cell lines. Since the C3 mAb recognizes the intracellular C-terminal domain of EphA2, full-length EphA2 and EphA2-CF could be distinguished based on their molecular weights. WB analysis revealed that the 135-kDa EphA2 protein was detected at varying levels in many HCC lines, with JHH4, JHH6, and SK-Hep1 cells expressing it at high levels. JHH2, SK-Hep1, Hep3B, and JHH6 cells expressed the active form (50-kDa) of MT1-MMP. Therefore, the 60-kDa EphA2-CF fragment was clearly detected in SK-Hep1 cells and faintly detected in JHH6 cells, consistent with the presence of MT1-MMP (Figs. 1A and S1). To further investigate whether inflammatory stimuli regulate EphA2 processing, SK-Hep1 cells were treated with IL-1β. IL-1β treatment increased EphA2 expression, MT1-MMP activation, and EphA2-CF production in a time-dependent manner (Fig. S2), suggesting that inflammatory signals in the tumor microenvironment may promote EphA2 cleavage. Furthermore, to confirm the subcellular location of EphA2 and EphA2-CF, biotinylation followed by streptavidin-based extraction of membrane proteins was performed in JHH6 and SK-Hep1 cells. WB confirmed the presence of EphA2 and EphA2-CF on the membranes of HCC cells (Fig. 1B).

### HCC cells expressing MT1-MMP and EphA2 are insensitive to growth suppression in the presence of EA1 stimulation

To investigate the functional impact of EphA2-CF expression on cell proliferation and malignant progression, we compared the effects of EA1 stimulation on HCC cell growth in JHH4 and SK-Hep1 cells, which differ in MT1-MMP expression. SK-Hep1 cells, which express EphA2-CF, were resistant to EA1-mediated growth inhibition by EphA2, whereas JHH4 cells, which do not produce EphA2-CF, exhibited EA1-mediated growth suppression (Fig. 1C, D). Therefore, to examine the role of MT1-MMP in the promotion of HCC cell growth in the presence of EA1, MT1-MMP expression was silenced in SK-Hep1 cells using shRNA. MT1-MMP knockdown significantly reduced EphA2-CF protein production (Fig. 1E) and restored sensitivity to EA1-induced growth suppression (Fig. 1F). These results suggest that EphA2-CF produced by MT1-MMP cleavage can enhance cell proliferation even in the presence of EA1.

### EphA2-CFs are resistant to the EA1-induced inhibition of growth and survival

**Fig. 2:**
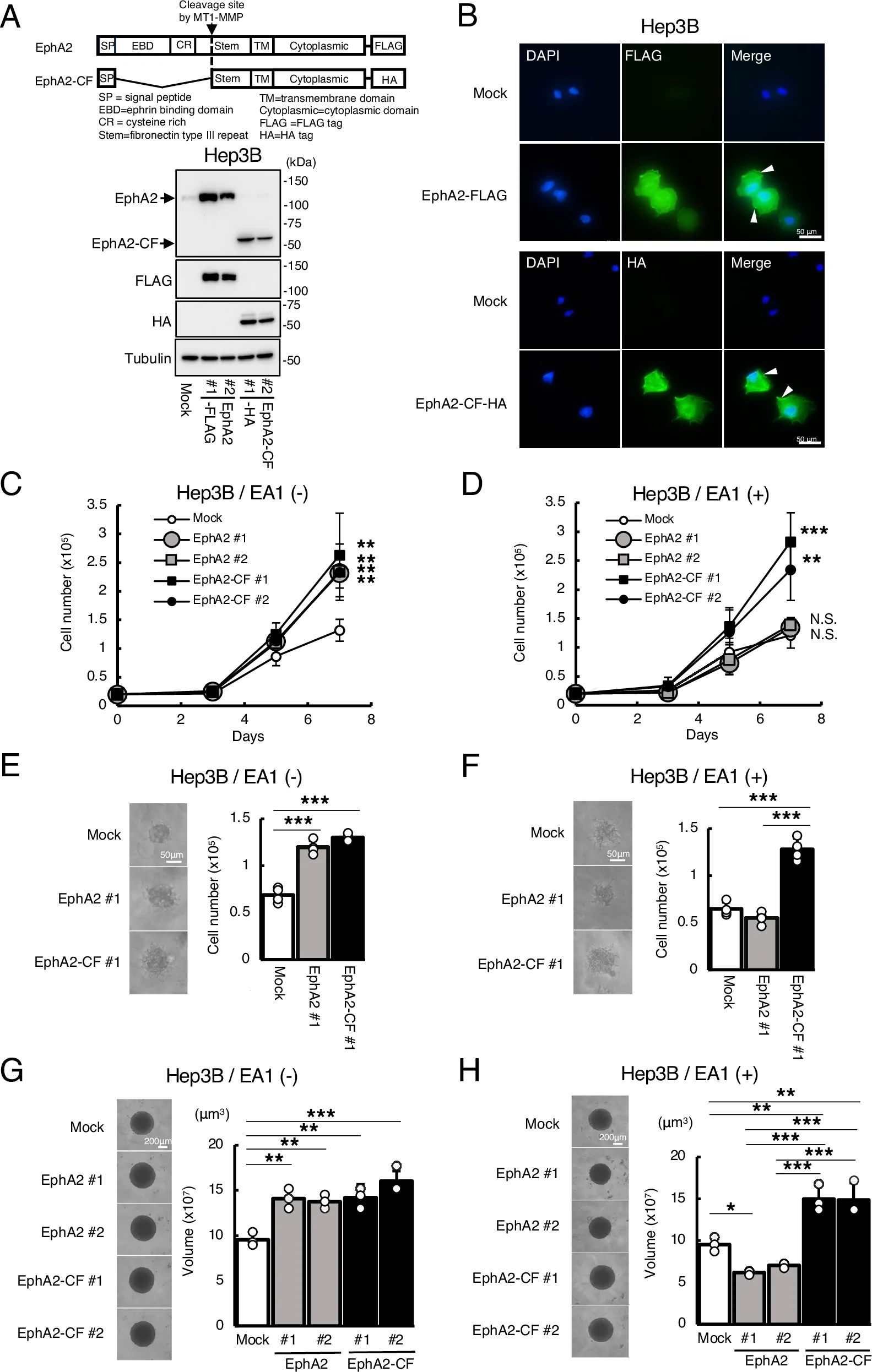
EphA2-CF is resistant to growth and survival inhibition induced by EA1 stimulation. **A** Schematic diagram of EphA2 and EphA2-CF molecules. EphA2-CF lacks the ligand-binding domain (LBD). WB analysis of enforced EphA2-Flag and EphA2-CF-HA proteins in Hep3B cells using anti-EphA2 (C-3), anti-FLAG, and anti-HA monoclonal antibodies (mAbs). **B** Representative immunofluorescence images showing DAPI staining of EphA2-FLAG, and EphA2-CF-HA in Hep3B cells expressing EphA2 or EphA2-CF. The white arrows show the expression of EphA2 and EphA2-CF: Scale bars, 50 µm. **C, D** Cell proliferation analysis of the cells described in (**A**) in the presence or absence of EA1. Cell counts were performed after incubation for 3, 5, and 7 days. **E, F** Effect of enforced EphA2 or EphA2-CF expression on cell proliferation in Hep3B cells under three-dimensional collagen gel culture with 10% FBS in the presence or absence of EA1. Cell counts were performed after 10 days of incubation: Scale bars, 50 µm. **G, H** Effect of enforced EphA2 or EphA2-CF expression on spheroid growth in Hep3B cells in the presence or absence of EA1. Spheroid volumes were measured after 12 days of incubation: Scale bars, 200 µm. All experiments were performed in duplicate or triplicate, and three independent experiments were conducted. Bar graphs represent data from at least three independent experiments. Statistical analysis was performed using one-way ANOVA with Tukey multiple comparisons test. Data are presented as mean ± SD (**C–H**) (*P < 0.05, **P < 0.01, ***P < 0.001, N.S., not significant).

Although previous studies have shown that EphA2 cleavage promotes malignant phenotypes, the mechanism by which EphA2-CF contributes to tumor cell growth and survival remains unclear. To understand this, we stably expressed EphA2 and EphA2-CF in Hep3B cells that do not express endogenous EphA2 and examined their effects. Western blotting confirmed the stable expression of FLAG-tagged EphA2, and HA-tagged EphA2-CF in Hep3B cells (Fig. 2A), and immunocytochemistry verified their membrane localization (Fig. 2B, white arrows). Under EGF-containing culture conditions (10% FBS), 2-dimensional (2-D), 3-D growth, and spheroid formation were performed with or without EA1 stimulation (Fig. 2C–H). Without EA1 stimulation, EphA2- and EphA2-CF-expressing Hep3B cells showed significantly increased cell proliferation and survival compared with mock-transfected controls (Figs. 2C, E, G and S3A). Meanwhile, EA1 stimulation suppressed growth and survival in EphA2-expressing cells but did not affect EphA2-CF-expressing cells (Fig. 2D, F, H and S3B).

### EphA2-S^897^ phosphorylation is critical for EphA2-CF-induced cell growth and survival in the presence of EA1 stimulation

**Fig. 3:**
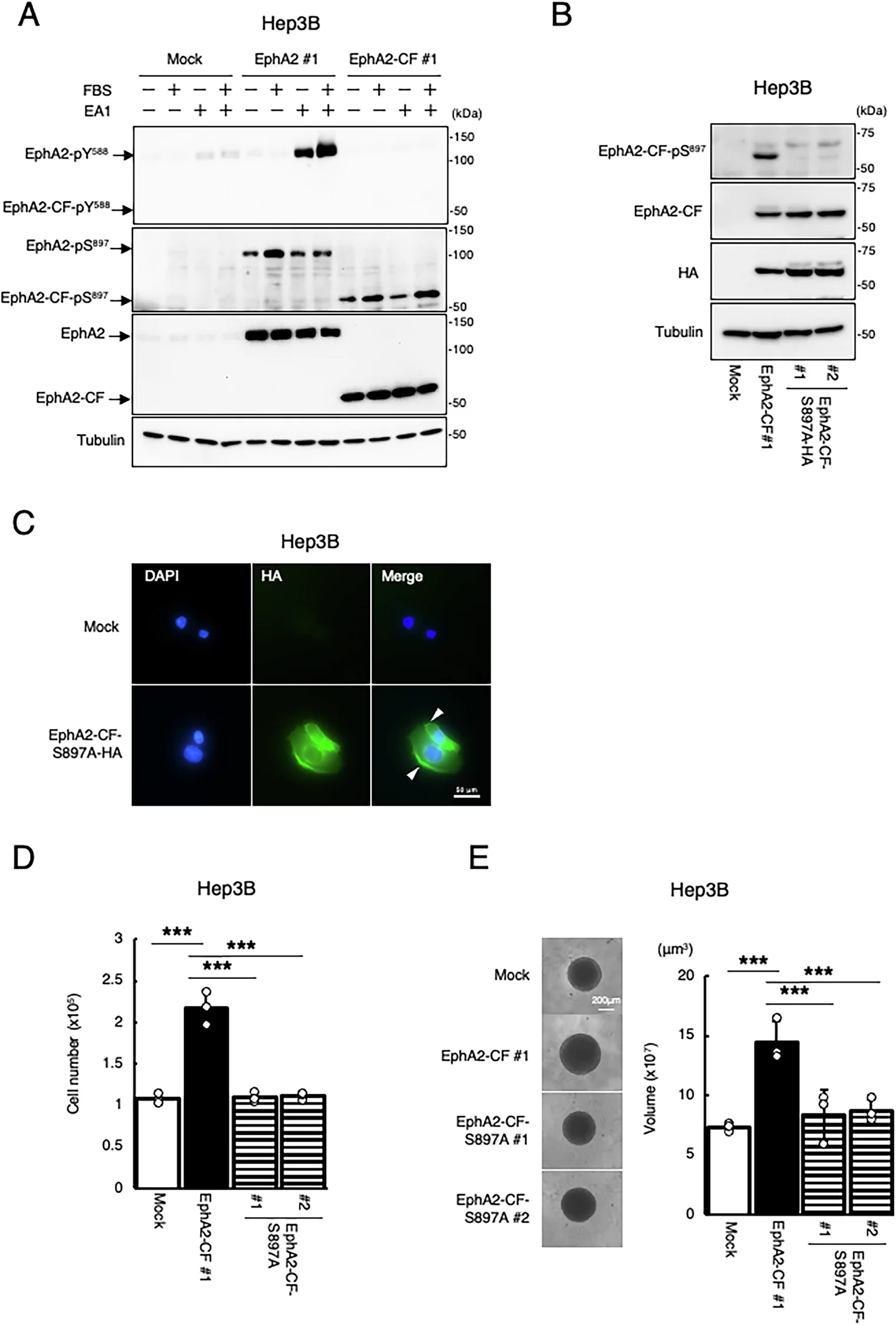
EphA2-CF-S^897^ phosphorylation is critical for EphA2-CF-induced growth and survival of HCC cells in the presence of EA1 stimulation. **A** Detection of EphA2 expression, EphA2-Y^588^ and -S^897^ phosphorylation in Hep3B cells expressing EphA2 or EphA2-CF (EphA2 #1, EphA2-CF #1) treated with or without 10% FBS and EA1. β-tubulin was used as an internal control. **B** WB analysis of enforced EphA2-CF and EphA2-CF-S897A expression in Hep3B cells using anti-EphA2-S^897^ phosphorylation, anti-EphA2 (C-3), and anti-HA mAbs. β-tubulin was used as an internal control. **C** Representative immunofluorescence images showing DAPI staining and localization of HA in Hep3B cells overexpressing EphA2-CF-S897A. White arrows indicate the localization of EphA2-CF-S897A: Scale bars, 50 µm. **D** Cell proliferation analysis was conducted by hemocytometer counting. Cell counts were performed after 5 days of incubation. **E** Effect of enforced EphA2-CF and EphA2-CF-S897A expression on spheroid growth in Hep3B cells. Spheroid volume was measured after 12 days of incubation. Scale bars, 200 µm. All experiments were performed in triplicate, and three independent experiments were conducted. Bar graphs represent data from at least three independent experiments. Statistical analysis was performed using one-way ANOVA with Tukey’s multiple comparisons test. Data are presented as means ± SD (**D, E**) (***P < 0.001).

To investigate the signaling pathways involved in EphA2-induced cell growth and survival, we analyzed the EphA2-dependent tumor-suppressive (EphA2-Y^588^ phosphorylation) and oncogenic (EphA2-S^897^ phosphorylation) signaling pathways. Under serum starvation, EphA2- or EphA2-CF‒expressing cells exhibited partial autophosphorylation at EphA2-S^897^, which was further upregulated upon treatment with 10% FBS (Fig. 3A). Although EA1 stimulation induced EphA2-Y^588^ phosphorylation in EphA2-expressing cells, resulting in reduced phosphorylation of S^897^, cells expressing EphA2-CF did not exhibit EphA2-Y^588^ phosphorylation in response to EA1 (Fig. 3A). Next, to investigate whether EphA2-S^897^ phosphorylation contributes to EphA2-CF-dependent cell growth and survival, EphA2-CF-S897A, a dominant-negative mutant for EphA2-CF-S^897^ phosphorylation, was introduced into Hep3B-expressing EphA2-CF (Fig. 3B). Expression and membrane localization of the mutant protein was confirmed by immunocytochemistry (Fig. 3C). Cell growth and spheroid formation assays showed that EphA2-CF-induced cell growth and survival were completely reduced compared with those in the mock group (Figs. 3D, E and S4). These results suggest that EphA2-CF constitutively activates EphA2-S^897^ phosphorylation even in the presence of EA1 stimulation, contributing to the promotion of HCC cell growth and survival.

### Quantitative analysis of cell signaling pathways that regulate EphA2-CF–induced cell growth and survival

**Fig. 4:**
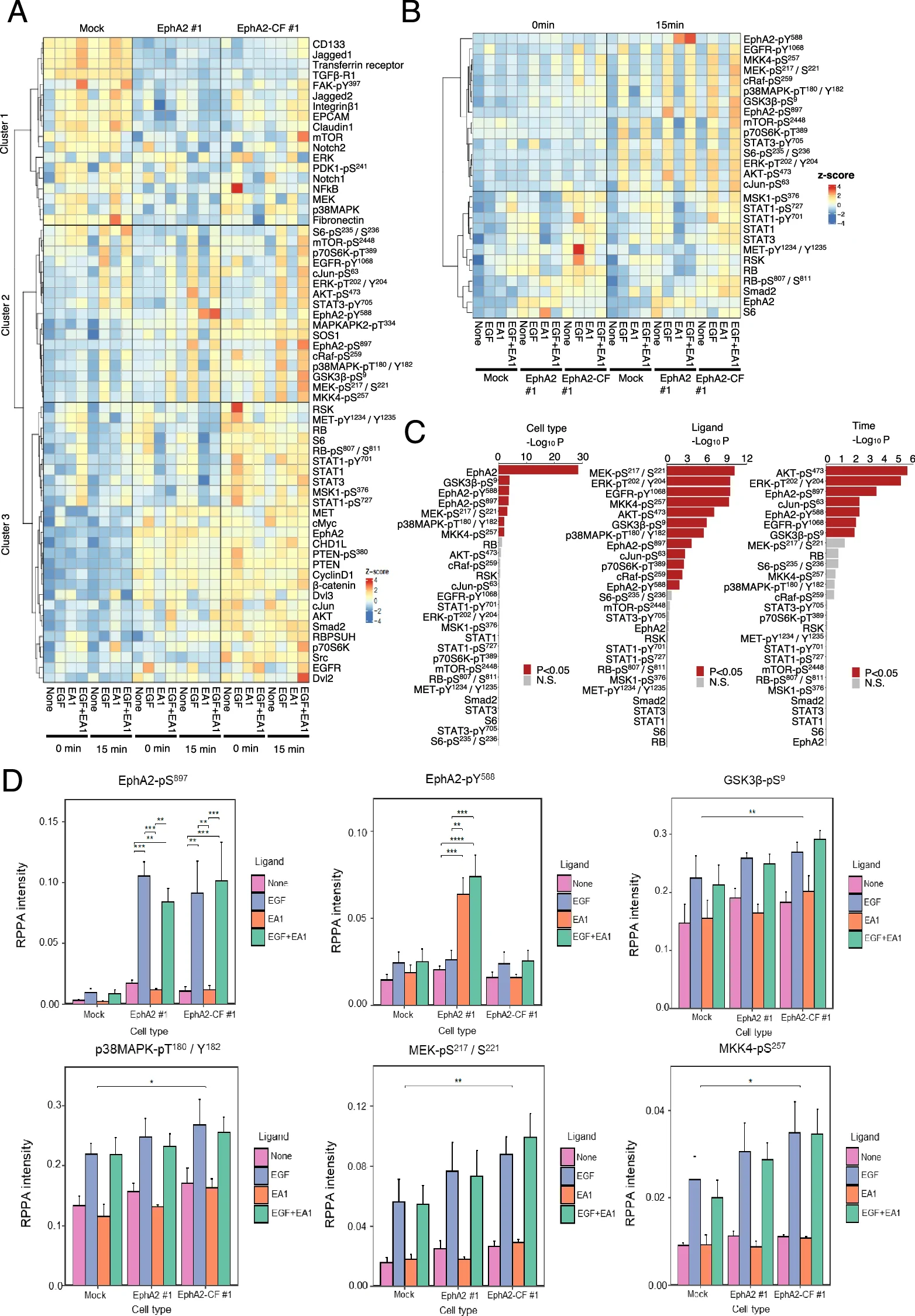
Quantitative analysis of cell signaling pathways and crosstalk that regulate EphA2-CF-induced growth and survival in HCC cells using reverse phase protein array (RPPA). **A** RPPA analysis of 62 cancer-related signaling molecules was performed in Hep3B cells (Mock, EphA2, EphA2-CF-expressing cells) to examine their expression and phosphorylation in response to EGF (20 ng/mL) and EA1 (1.0 µg/mL). **B** RPPA analysis of protein expression and phosphorylation in response to EGF and EA1 stimulation, focusing on signaling molecules with high activity in EphA2-CF cells within Clusters 2 and 3. Hierarchical clustering of the data revealed two distinct clusters: proteins and phosphorylated proteins, showing higher levels of phosphorylation after 15 min of EGF or EGF + EA1 stimulation, and proteins and phosphorylated proteins, showing no significant differences in activity between 0 and 15 min of EGF or EGF + EA1 stimulation. **C** A three-way ANOVA without interaction for each protein or phosphoprotein to examine the effects of cell type (Mock, EphA2, and EphA2-CF), ligand (None, EGF, EA1, and EGF + EA1), and time (0 and 15 min). **D** Further analysis of data for these six phosphoproteins after 15 min of stimulation using a two-way ANOVA to assess the effects of cell type and ligand.

Using Hep3B cells expressing Mock, EphA2, and EphA2-CF, we examined the expression and phosphorylation of 62 cancer-related signaling molecules in response to EGF and EA1 using reverse phase protein array (RPPA) analysis. Hierarchical clustering of RPPA data revealed four distinct clusters (Fig. 4A). Cluster 1 consisted of proteins and phosphoproteins whose activities were reduced in cells expressing EphA2 and EphA2-CF. Cluster 2 included phosphoproteins involved in the ERK/MAPK and PI3K/AKT signaling pathways. Cluster 3 consisted of proteins and phosphorylated proteins preferentially activated in EphA2-CF-expressing cells alone or in combination with EphA2.

Next, we focused on clusters 2 and 3 and repeated the RPPA analysis following 0 and 15 min of stimulation with EGF, EA1, or EGF plus EA1. Hierarchical clustering of the data revealed two distinct clusters: phosphoproteins with higher phosphorylation after 15 min of stimulation, and proteins and phosphoproteins with minimal changes at 0 and 15 min of stimulation (Fig. 4B). A three-way analysis of variance (ANOVA) without interaction was performed for each protein or phosphoprotein to examine the effects of cell type (Mock, EphA2, and EphA2-CF), ligand (none, EGF, EA1, and EGF + EA1), and time (0 and 15 min). All three main effects were significant for the phosphorylation of EphA2-S^897^, EphA2-Y^588^, and GSK3β-S^9^. The main effects of cell type and ligand were significant for the phosphorylation of MEK-S^217/221^, MKK4-S^257^, and p38MAPK-T^180^/Y^182^ (Fig. 4C).

Phosphoprotein levels between cell type and ligand, analyzed via two-way ANOVA after 15 min of stimulation (Fig. 4D), revealed significant interactions only for the phosphorylation of EphA2-S^897^ (*F* (6,24) = 4.025, *p* = 0.006) and EphA2-Y^588^ (*F* (6,24) = 5.246, *p* = 0.001). For EphA2-S^897^ implantation, the simple main effect of cell type was significant for EGF (*F* (2,24) = 15.707, *p* < 0.0001) and EGF + EA1 (*F* (2,24) = 14.227, *p* < 0.0001), whereas the simple main effect of the ligand was significant for EphA2 (*F* (3,24) = 13.144, *p* < 0.0001) and EphA2-CF (*F* (3,24) = 14.295, *p* < 0.0001). Pairwise comparisons indicated that enhanced EGF-induced EphA2-S^897^ phosphorylation occurred in cells expressing EphA2 and EphA2-CF. Regarding the phosphorylation of EphA2-Y^588^, the simple main effect of cell type was significant for EA1 (*F* (2,24) = 17.558, *p* < 0.0001) and EGF + EA1 (*F* (2,24) = 19.264, *p* < 0.0001), and the simple main effect of ligand was significant for EphA2 (*F* (3,24) = 17.388, *p* < 0.00001). Pairwise comparisons indicated that enhanced EA1-induced phosphorylation of EphA2-Y^588^ occurred in cells expressing EphA2. These results demonstrate that cells expressing EphA2 exhibit both ligand-independent (EphA2-S^897^ phosphorylation) and ligand-dependent (EphA2-Y^588^ phosphorylation) phosphorylation of EphA2, whereas EphA2-CF cells only exhibit ligand-independent EphA2 phosphorylation. For the remaining four phosphoproteins, significant main effects were also observed for cell type (phosphorylation of MEK-S^217/221^: *F* (2,30) = 5.238, *p* = 0.011; MKK4-S^257^: *F* (2,30) = 3.506, *p* = 0.043; p38MAPK-T^180^/Y^182^: *F* (2,30) = 4.051, *p* = 0.028; GSK3β-S^9^: *F* (2,30) = 5.244, *p* = 0.011) and ligand (phosphorylation of MEK-S^217/221^: *F* (3,30) = 23.765, *p* < 0.0000001; MKK4-S^257^: *F* (3,30) = 23.72, *p* < 0.0000001; p38MAPK-T^180^/Y^182^: *F* (3,30) = 20.319, *p* < 0.000001; GSK3β-S^9^: *F* (3,30) = 11.741, *p* < 0.0001). Pairwise comparisons indicated higher phosphorylation of MEK, MKK4, p38 MAPK (activation), and GSK3β-S^9^ (inactivation) in cells expressing EphA2-CF than in Mock cells.

### Effects of MEK and p38 MAPK inhibition and GSK3β activation on EphA2-CF-induced Hep3B cell growth

**Fig. 5:**
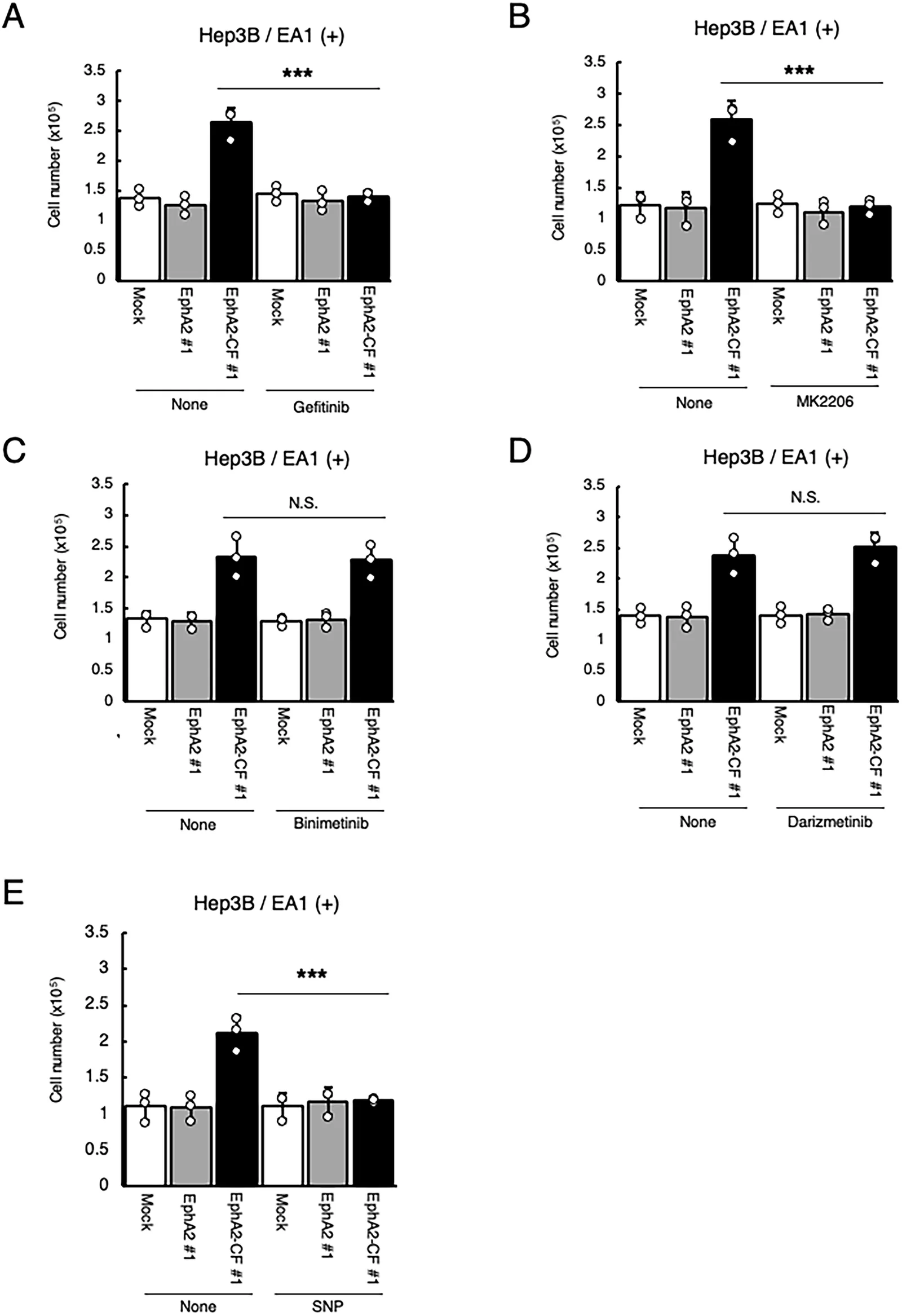
EGFR inhibitors, AKT inhibitors, and GSK3β activators suppress EphA2-CF-induced cell proliferation even in the presence of EA1 stimulation. Effects of EGFR, AKT, MEK, MKK4, and p38 MAPK inhibitors and GSK3β activator on EphA2-CF-induced Hep3B cell growth in the presence of EA1 (1.0 µg/ml). Cells were treated with EGFR inhibitor gefitinib (5.0 μM) (**A**), AKT inhibitor MK2206 (1.0 μM) (**B**), MEK inhibitor binimetinib (1.0 μM) (**C**), MKK4 and p38MAPK inhibitor darizmetinib (1.0 μM) (**D**) and GSK3β activator sodium nitroprusside, (SNP; 0.1 mM) (**E**). All experiments were performed in duplicate, and three independent experiments were conducted. The bar graphs represent data from at least three independent experiments. Statistical analysis was performed using one-way ANOVA with Tukey’s multiple comparison test. Data are presented as mean ± SD (***P < 0.001, N.S., not significant).

**Fig. 6:**
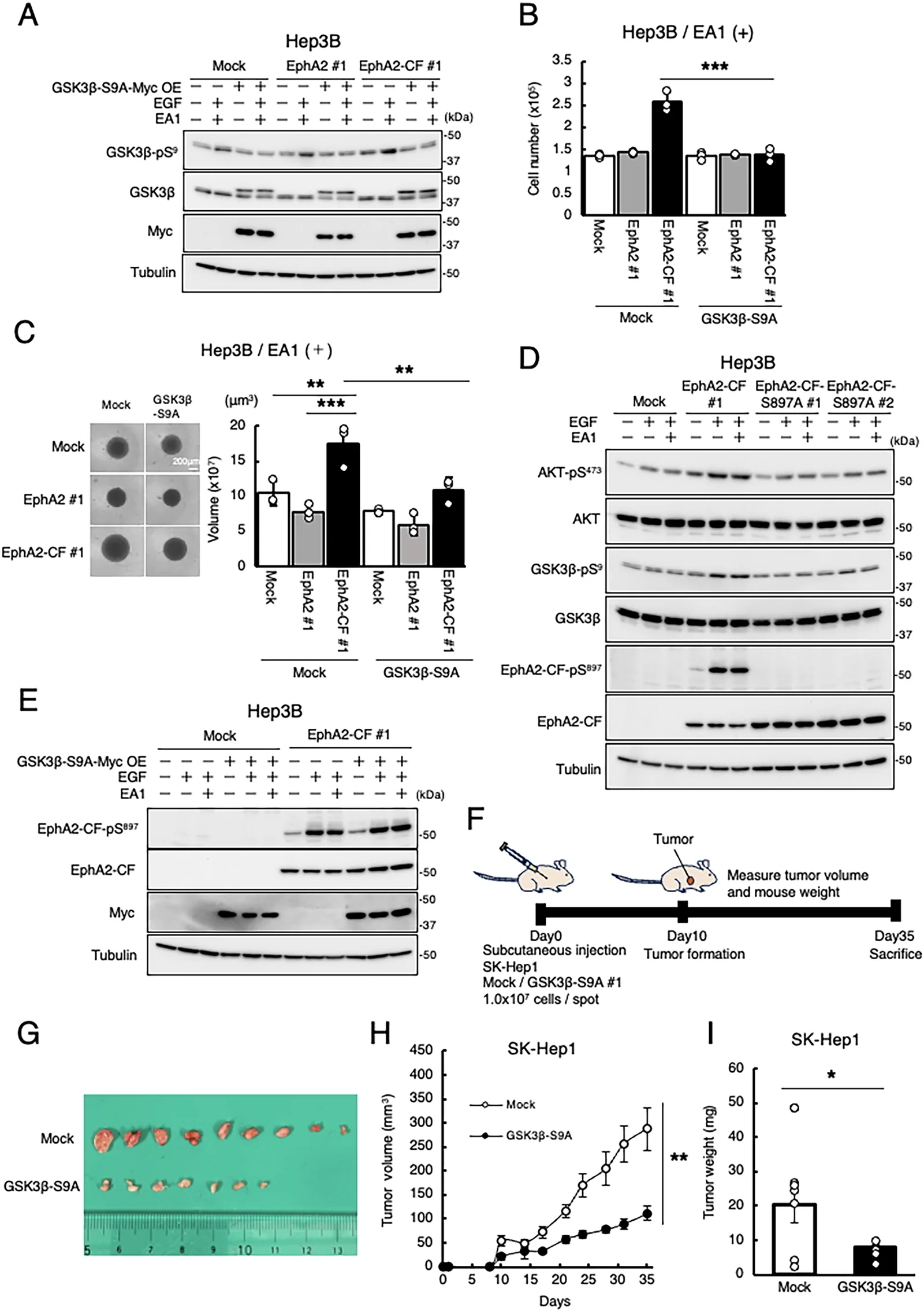
The EGFR/EphA2-CF/AKT/GSK3β axis is a key pathway involved in EphA2-CF-induced cell proliferation and survival, even in the presence of EA1 stimulation. **A** Effect of GSK3β-S9A expression on GSK3β-S^9^ phosphorylation in Hep3B cells expressing Mock, EphA2, or EphA2-CF and treated with or without EGF and EA1. The GSK3β-S^9^ phosphorylation was detected through WB using its specific mAb. **B** Cell proliferation analysis was conducted using a hemocytometer in Hep3B cells expressing Mock, EphA2, or EphA2-CF after transfection of GSK3βS9A-Myc in the presence of EA1. **C** Effect of GSK3β-S9A-Myc expression on spheroid growth in Hep3B cells expressing Mock, EphA2, or EphA2-CF after transfection of GSK3βS9A-Myc in the presence of EA1. **D** Effect of EphA2-CF-S897A expression on EphA2-CF-S^897^, AKT-S^473^, and GSK3β-S^9^ phosphorylation in Hep3B cells expressing Mock or EphA2-CF in the presence of EGF and EA1. **E** Effect of GSK3β-S9A expression on EphA2-CF-S^897^ phosphorylation in Hep3B cells expressing mock or EphA2-CF in the presence of EGF and EA1. All experiments were carried out at a concentration of 20 ng/ml of EGF and 1 µg/ml of EA1. All experiments were performed in triplicate, and three independent experiments were conducted. The bar graphs represent data from at least three independent experiments. **F** Schematic diagram of the experimental design using a mouse xenograft model. Mock or GSK3β-S9A-expressing SK-Hep1 cells (1.0 × 10^7^ cells) were subcutaneously injected into mice (6 mice/group, 2 spots/mouse). **G** Comparison of xenograft tumors resected from BALB/c nude mice (5 weeks after tumor subcutaneous injection). Tumor growth curve (**H**) and weight (**I**) of GSK3β-S9A-expressing SK-Hep1 xenografts in BALB/c nude mice. Statistical analysis was performed using one-way ANOVA with Tukey’s multiple comparisons test. Data are presented as mean ± SD (**B, C**) (**P < 0.01, ***P < 0.001).

To verify the involvement of these signaling molecules in EphA2-CF-induced cell proliferation under EA1 stimulation, we tested inhibitors of EGFR, MEK, MKK4/p38 MAPK [27], and AKT, as well as GSK3β activators. Inhibition of EGFR with gefitinib suppressed EphA2-CF-induced cell proliferation (Fig. 5A), as did inhibition of AKT with MK2206 (Fig. 5B). However, inhibition of MEK and MKK4/p38 MAPK with binimetinib and darizumetinib, respectively, while suppressing the phosphorylation of their respective targets, did not affect EphA2-CF-induced cell proliferation (Figs. 5C, D and S5A, B). Interestingly, treatment of cells with SNP, which reduces phosphorylation of GSK3β at S^9^, can inhibit EphA2-CF-induced cell proliferation (Figs. 5F and S5C). These results demonstrate that EphA2-CF-induced cell proliferation is mediated by EGFR/PI3K/AKT and their downstream GSK3β-S^9^ signaling pathways. To further demonstrate that GSK3β-S^9^ phosphorylation is responsible for EphA2-CF-induced cell proliferation, we transfected a dominant-negative mutant of GSK3β-S9A into EphA2-CF-expressing cells. Cells expressing GSK3β mutant GSK3β-S9A exhibited reduced GSK3β-S^9^ phosphorylation (Fig. 6A) and significantly suppressed cell proliferation and spheroid formation (Figs. 6B, C and S6). Furthermore, cells expressing EphA2-CF-S897A exhibited suppressed AKT phosphorylation and significantly reduced GSK3β-S^9^ phosphorylation (Fig. 6D), while expression of GSK3β-S9A mutant did not affect EphA2-CF phosphorylation at S^897^ (Fig. 6E).

To demonstrate that EphA2-CF promotes malignant potential through the inactivation of GSK3β in vivo, we transfected SK-Hep1 cells, which endogenously produce EphA2-CF, with the active GSK3β-S9A mutant and assessed their proliferation ability and tumorigenicity in mice following subcutaneous implantation (Figs. 6F and S7A). The GSK3β-S9A mutant-transduced cells exhibited significantly reduced growth activity (Fig. S7B). In addition, mice were subcutaneously implanted with 1 × 10^7^ GSK3β-S9A mutant-transduced cells and sacrificed 35 days later; tumorigenicity was compared with that in mock mice (Fig. 6F). The GSK3β-S9A mutant-transduced cells, which activated GSK3β, exhibited significantly reduced tumorigenicity and tumor weight compared with mock cells (Fig. 6G–I).

In addition to the results for HCC cells, in vivo analysis using the Cancer Proteome Atlas (TCPA) database from HCC tissues showed a positive correlation between EphA2-S^897^ and GSK3β-S^9^ phosphorylation (r = 0.541) (Fig. S8A). Furthermore, the AKT-S^473^ phosphorylation correlated with EphA2-S^897^ or GSK3β-S^9^ (r = 0.639 or 0.690, respectively) (Fig. S8B, C). Taken together, these in vitro and in vivo results suggest that the EGFR/EphA2-CF/AKT/GSK3β axis promotes HCC cell proliferation and survival even in the presence of EA1 stimulation.

### EphA2-NF evades EA1-dependent tumor suppression by acting as a decoy receptor

**Fig. 7:**
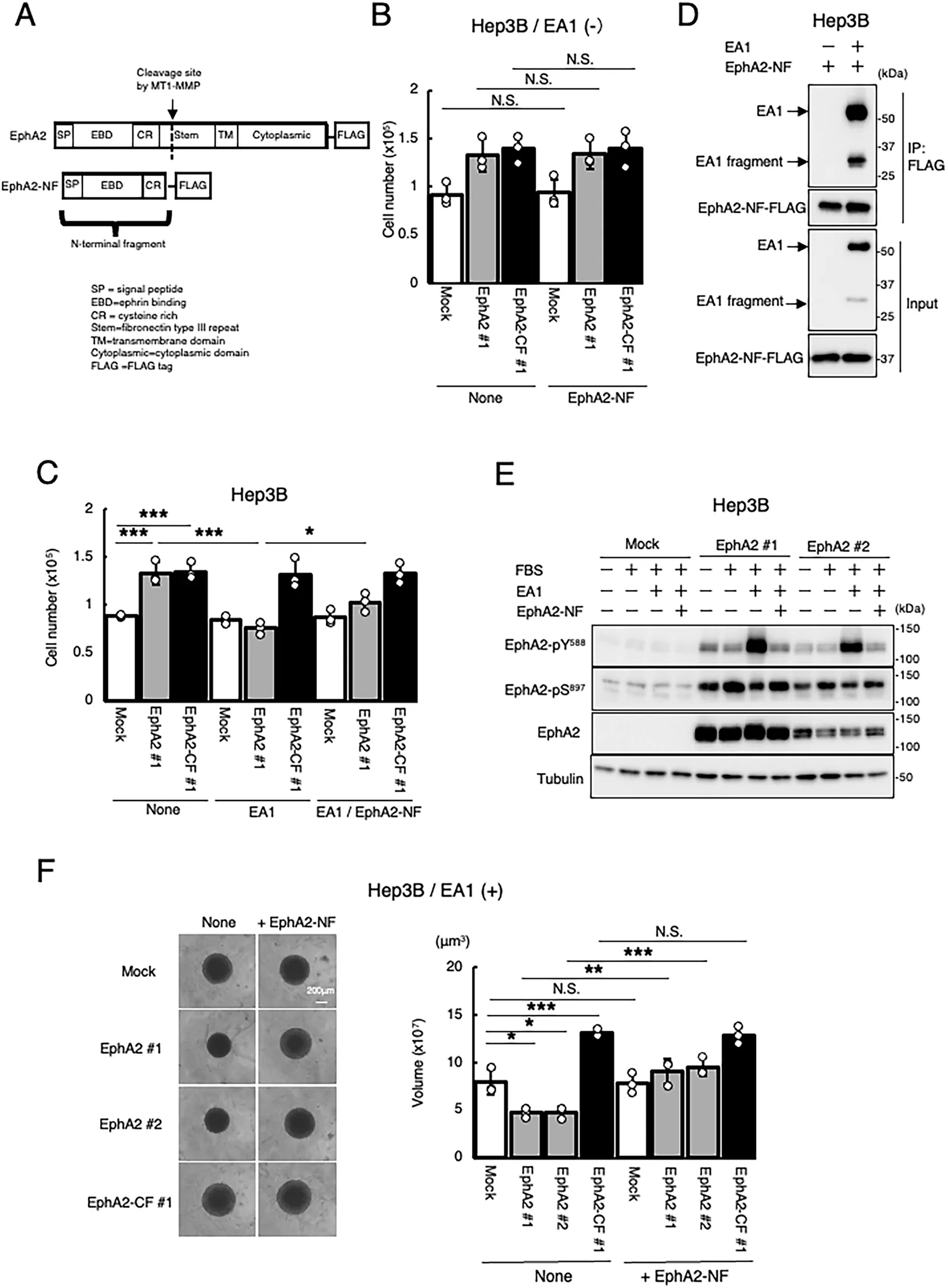
EphA2-NF attenuates EA1-dependent tumor suppression by acting as a Decoy receptor. **A** Structure for recombinant EphA2 and EphA2-NF proteins. **B** Proliferation analysis of Hep3B cells cultured in 24-well plates in the absence of EphA2-NF. **C** Pull-down analysis of the Interaction between soluble EphA2-NF and EA1 protein. **D** Effect of EphA2-NF on EA1-induced growth inhibition in Hep3B cells expressing EphA2 or EphA2-CF in the presence of EA1. **E** Effect of EphA2-NF on EphA2-Y^588^ and -S^897^ phosphorylation. β-tubulin was used as an internal control. **F** Effect of EphA2-NF on EA1-induced spheroid growth inhibition in Hep3B cells expressing EphA2 or EphA2-CF in the presence of EA1. All experiments were carried out at a concentration of 5 µg/ml of EphA2-NF and 1 µg/ml of EA1. All experiments were performed in duplicate or triplicate, and three independent experiments were conducted. The bar graphs represent data from at least three independent experiments. Statistical analysis was performed using one-way ANOVA with Tukey’s multiple comparison test. Data are presented as mean ± SD (**A, B, E**) (*P < 0.05, **P < 0.01, ***P < 0.001, N.S., not significant).

EphA2-NF, released after cleavage of EphA2, is a robust biomarker of early pancreatic cancer. To understand its functional role, we evaluated the effect of recombinant EphA2-NF protein on 2-D cell growth using Hep3B cells expressing EphA2 or EphA2-CF. Our results showed that treatment with EphA2-NF alone did not affect cell proliferation in HCC cells expressing Mock, EphA2, or EphA2-CF. (Fig. 7A, B). However, co-treatment with EphA2-NF and EA1 inhibited EA1-induced growth inhibition in EphA2-expressing cells, but not in cells expressing EphA2-CF (Fig. 7C, S9).

To assess the interaction between soluble EphA2-NF and EA1 proteins, FLAG-tagged EphA2-NF was immunoprecipitated with a FLAG-tag mAb. EphA2-NF coprecipitated with EA1 (Fig. 7D), suggesting that EphA2-NF competes with full-length EphA2 for the binding of EA1. In fact, it suppressed the phosphorylation of EphA2-Y^588^ induced by EA1 and further inhibited the decrease in EphA2-S^897^ (Fig. 7E). Based on these results, we determined that EphA2-NF acts as a decoy receptor for EA1, thereby compromising the inhibition of spheroid growth by EA1 (Figs. 7F and S9). These results suggest that the released EphA2-NF functions as a decoy receptor for EA1, promoting cancer cell proliferation via the suppression of EA1 signaling from unprocessed EphA2 on the cell surface.

## Discussion

In our previous study, we demonstrated that MT1-MMP–dependent processing of EphA2 plays a central role in malignant progression by generating distinct cleavage fragments [16]; however, the molecular mechanisms through which the resulting fragments regulate malignancy remain unclear. In the present study, we clarified that MT1-MMP–mediated cleavage produces two functionally distinct products, EphA2-CF and EphA2-NF, which cooperatively enhance malignant phenotypes through different signaling mechanisms. Furthermore, the expression of both MT1-MMP and EphA2 is known to be induced by inflammatory cytokines [28, 29], suggesting that not only cancer cells but also the inflammatory tumor microenvironments may enhance EphA2 processing. Together, they constitute a previously unrecognized dual regulatory system that amplifies oncogenic signaling and suppresses tumor-inhibitory pathways within the tumor microenvironment.

**Fig. 8:**
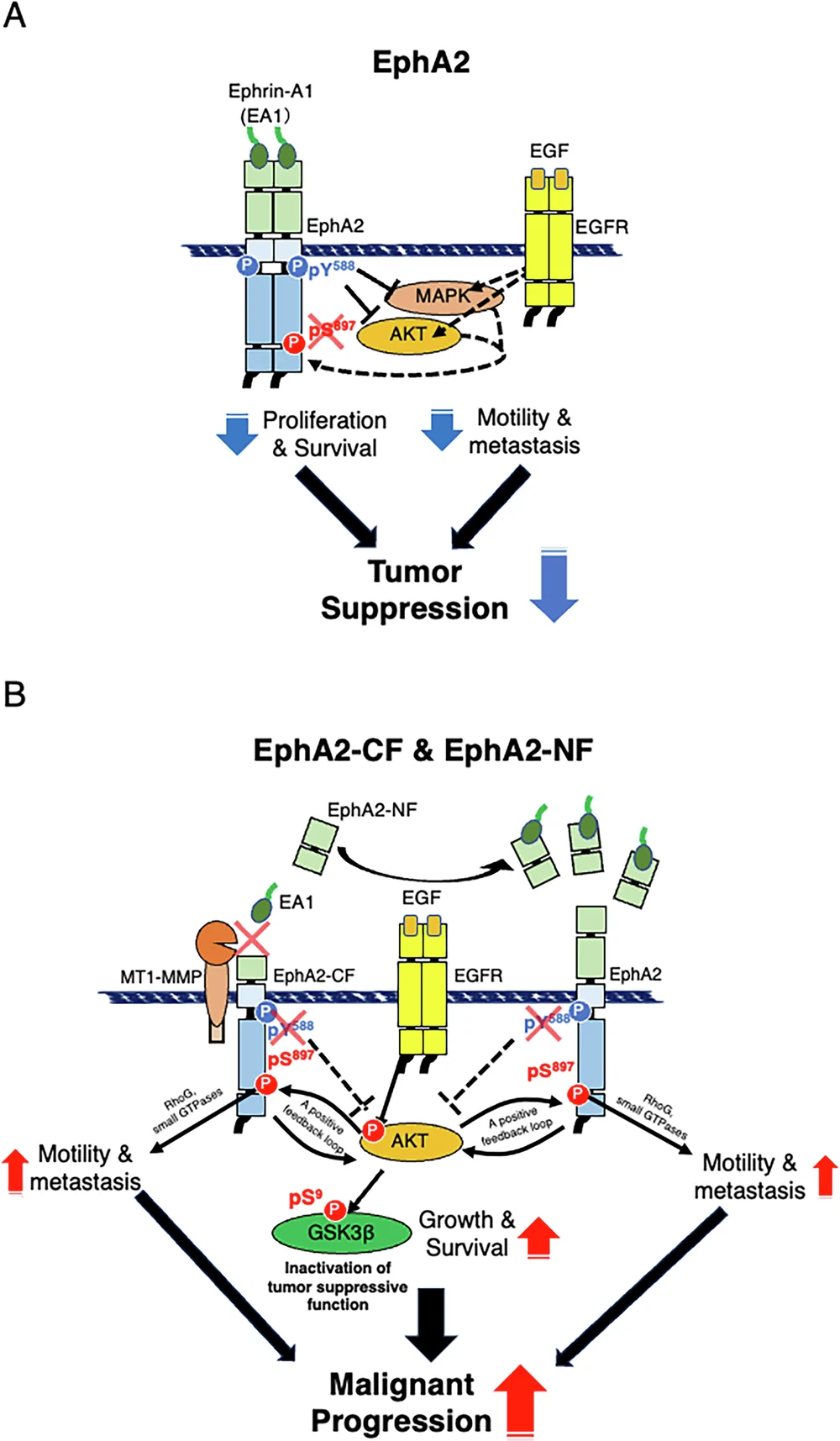
Schematic diagram of the different roles of EphA2 fragments in the acquisition of malignant phenotypes in HCC cells. **A** In the absence of MT1-MMP expression, EphA2 is stimulated by EA1 and suppresses downstream EGFR signaling through a ligand-dependent tumor suppressor signal. **B** In cancer cells expressing MT1-MMP, EphA2 is processed by MT1-MMP to produce two proteolytic fragments: EphA2-NF and EphA2-CF. EphA2-NF is released from the cell membrane and acts as a decoy receptor that traps EA1 on the cell surface, allowing wild-type EphA2 to avoid stimulation by the ligand. Furthermore, EphA2-CF enhances the phosphorylation of EphA2-S^897^ even in the presence of EA1, thereby promoting proliferation and survival via the phosphorylation of AKT-S^473^ and GSK3β-S^9^.

Mechanistically, EphA2-CF is unresponsive to EA1 and remains stably expressed on the plasma membrane [16]. While ligand-bound EphA2 undergoes EphA2-Y^588^ phosphorylation and suppresses EphA2-S^897^ phosphorylation downstream of EGFR/AKT signaling, EphA2-CF lacks the N-terminal ligand-binding domain and thus cannot interact with EA1. Consequently, EphA2-S^897^ phosphorylation acts as a ligand-independent oncogenic signal and promotes activation of the small GTPase RhoG, leading to malignant progression [16, 30, 31] (Fig. 8). EphA2-S^897^ phosphorylation has been reported to promote invasion or motility through different downstream signals, such as EGFR/MEK/RSK, across tumor types [32, 33]. However, our data from HCC cells indicate that MEK inhibition does not affect EphA2-CF-induced proliferation, suggesting that the aforementioned pathways are not involved in EphA2-CF-driven proliferation in HCC cells (Fig. 5C). Therefore, EphA2-CF does not uniformly activate all downstream pathways but selectively engages signaling pathways depending on the cellular context.

Using RPPA analysis, we further identified the AKT/GSK3β axis as a novel downstream pathway of EphA2-CF. EphA2-CF-expressing cells exhibited increased phosphorylation of AKT-S^473^ and GSK3β-S^9^, which was accompanied by enhanced proliferative capacity. Importantly, introduction of a dominant-negative GSK3β-S9A mutant or pharmacological inhibition of AKT markedly reduced EphA2-CF-dependent proliferation and decreased GSK3β-S^9^ phosphorylation. These results demonstrate that EphA2-CF drives proliferation through the AKT/GSK3β signaling cascade. Notably, phosphorylation of GSK3β at S^9^ inactivates its tumor-suppressive function and promotes β-catenin stabilization, a mechanism known to enhance tumorigenic potential in HCC (Fig. 6G, H, I) [34, 35]. Correlative analysis using the TCPA HCC dataset further revealed a strong positive relationship between EphA2-S^897^ and GSK3β-S^9^ phosphorylation (r = 0.541; Fig. S8A), supporting a mechanistic link between these two pathways.

Previous studies have reported that phosphorylation of EphA2-S^897^ can activate AKT through the EphA2/Ephexin-4/RhoG complex [36–39]. Based on these findings and our current results, we propose a positive feedback loop in which AKT activation promotes further EphA2-S^897^ phosphorylation, which in turn enhances GSK3β-S^9^ phosphorylation (Fig. 8). This loop may stabilize β-catenin, driving uncontrolled proliferation and survival [34, 35]. The identification of this EphA2-CF–AKT/GSK3β feedback circuit provides a new conceptual framework for understanding how proteolytic processing of receptor tyrosine kinases contributes to sustained oncogenic signaling, independent of ligand engagement.

During MT1-MMP–mediated processing, the EphA2-NF is released while retaining EA1-binding ability [17]. Here, we demonstrate that EphA2-NF acts as a soluble decoy receptor that competes with membrane-bound EphA2 for EA1, thereby blocking ligand-induced tumor-suppressive signaling (Fig. 8). Serum detection of EphA2-NF in patients with pancreatic and gastric cancer supports its role as a circulating modulator of EA1 bioavailability [22, 40]. EphA2-NF therefore represents a previously unrecognized class of soluble decoy receptors that perturb ligand-receptor homeostasis within the tumor microenvironment.

Clinically, MT1-MMP-mediated EphA2 processing has been observed in multiple epithelial cancers, including breast, ovarian, skin and bladder cancers [15, 41–43]. Immunohistochemical analysis also revealed frequent EphA2 processing in intraductal papillary mucinous carcinomas derived from intraductal papillary mucinous neoplasms, suggesting that this event may also contribute to the early stages of pancreatic carcinoma [22]. Importantly, most commercially available antibodies used for EphA2 immunostaining recognize the C-terminal cytoplasmic region; thus, accumulation of EphA2-CF could be misinterpreted as “high EphA2 expression” in clinical samples. This suggests that previous reports correlating EphA2 overexpression with poor prognosis may, in fact, reflect EphA2-CF accumulation rather than unprocessed receptor upregulation. Therefore, structural epitope-specific detection systems are critical for distinguishing full-length and processed EphA2 in both research and diagnostic settings. Such a distinction would provide a more accurate pathological assessment.

In conclusion, our study reveals that MT1-MMP-dependent EphA2 processing drives cancer progression through dual mechanisms: activation of the EGFR/AKT/GSK3β axis by EphA2-CF and sequestration of EA1-mediated tumor suppression by EphA2-NF (Fig. 8). These findings identify EphA2 processing as a pivotal oncogenic event and highlight EphA2-CF as a promising therapeutic target. Future efforts to develop EphA2-CF-specific inhibitors or diagnostic assays based on structural variants could redefine molecular targeted strategies against EphA2-driven cancers.

## Supplementary information

Table S1

Table S2

Supplementary_Information

Supplementary Figures

Western blot original data

## Data Availability

The data generated during the current study are available from the corresponding author upon reasonable request.
