# Supplementary material for: Proteolytic EphA2 fragments cooperatively promote hepatocellular carcinoma progression
Source: Cell Death Dis. 2026 Jun 29;17(1):799. doi: 10.1038/s41419-026-09024-1 (PMC13575192; doi:10.1038/s41419-026-09024-1)
Supplement: Supplementary file 1 [file 41419_2026_9024_MOESM1_ESM.pdf]

Supplementary Table S1.

Primary antibodies used in western blotting

Primary antibodies				
**Antibody**	**Species**	**Supplier**	**Reference**	**Dilution**
EphA2	Mouse	Santa Cruz	sc-398832	1:1000
Phospho-EphA2-Y^{588}	Rabbit	Cell Signaling Technology	12677	1:1000
Phospho-EphA2-S^{897}	Rabbit	Cell Signaling Technology	6347	1:1000
Ephrin-A1	Rabbit	Santa Cruz	sc-911	1:1000
Phospho-AKT-S^{473}	Rabbit	Cell Signaling Technology	4060	1:1000
Pan-AKT	Mouse	Cell Signaling Technology	2920	1:1000
Phospho-GSK3β-S^{9}	Rabbit	Cell Signaling Technology	5558	1:2000
GSK3β	Rabbit	Cell Signaling Technology	12456	1:2000
Phospho-ERK1/2-$Thr^{202/Tyr204}$	Rabbit	Cell Signaling Technology	4370	1:2000
Phospho-MEK1/2-$S^{217/221}$	Rabbit	Cell Signaling Technology	9154	1:2000
Phospho-SEK1/MKK4-S^{257}	Rabbit	Cell Signaling Technology	4514	1:1000
Phospho-p38 MAPK-$T^{180/T182}$	Rabbit	Cell Signaling Technology	4511	1:1000
MT1-MMP	Mouse	House made	222-1D8	1:500
Transferrin receptor	Mouse	Thermo Fisher Scientific	136800	1:1000
FLAG	Mouse	MBL	M185-3L	1:1000
HA	Mouse	MBL	M180-3	1:1000
Myc	Mouse	MBL	M192-3	1:1000
β-tubulin	Mouse	DSHB	E7	1:5000
