# Supplementary material for: Proteolytic EphA2 fragments cooperatively promote hepatocellular carcinoma progression
Source: Cell Death Dis. 2026 Jun 29;17(1):799. doi: 10.1038/s41419-026-09024-1 (PMC13575192; doi:10.1038/s41419-026-09024-1)
Supplement: Supplementary file 2 [file 41419_2026_9024_MOESM2_ESM.pdf]

Supplementary Table S2.

Primary antibodies used in RPPA

Primary antibodies				
**Antibody**	**Species**	**Supplier**	**Reference**	**Dilution**
EphA2	Mouse	Santa Cruz	sc-398832	1: 250
Phospho-EphA2-Y^{588}	Rabbit	Cell Signaling Technology	12677	1: 250
Phospho-EphA2-S^{897}	Rabbit	Cell Signaling Technology	6347	1: 250
Phospho-EGFR -Y^{1068}	Rabbit	Cell Signaling Technology	3777	1: 250
EGFR	Rabbit	Cell Signaling Technology	4267	1: 250
Phospho-c-Raf-S^{259}	Rabbit	Cell Signaling Technology	9421	1: 250
Phospho-ERK1/2-T^{202}/Y^{204}	Rabbit	Cell Signaling Technology	4370	1: 250
ERK1/2	Mouse	Cell Signaling Technology	4696	1: 250
Phospho-AKT-S^{473}	Rabbit	Cell Signaling Technology	4060	1: 250
Pan-AKT	Mouse	Cell Signaling Technology	2920	1: 250
Phospho-MEK1/2- $S^{217/221}$	Rabbit	Cell Signaling Technology	9154	1: 250
MEK1/2	Rabbit	Cell Signaling Technology	8727	1: 250
Phospho-S6 Ribosomal Protein-$S^{235/236}$	Rabbit	Cell Signaling Technology	4858	1: 250
S6 Ribosomal Protein	Rabbit	Cell Signaling Technology	2217	1: 250
Phospho-p70 S6 Kinase-T^{389}	Rabbit	Cell Signaling Technology	9234	1: 250
p70 S6 Kinase	Rabbit	Cell Signaling Technology	2708	1: 250
Phospho-mTOR-S^{2448}	Rabbit	Cell Signaling Technology	5536	1: 250
mTOR	Rabbit	Cell Signaling Technology	2983	1: 250

Phospho-GSK-3β-S^{9}	Rabbit	Cell Signaling Technology	5558	1: 250
Phospho-PTEN-S^{380}	Rabbit	Cell Signaling Technology	9551	1: 250
PTEN	Rabbit	Cell Signaling Technology	9188	1: 250
Phospho-PDK1-S^{241}	Rabbit	Cell Signaling Technology	3438	1: 250
Phospho-p38 MAPK-T^{180}/Y^{182}	Rabbit	Cell Signaling Technology	4511	1: 250
p38 MAPK	Rabbit	Cell Signaling Technology	8690	1: 250
Phospho-SEK1/MKK4-S^{257}	Rabbit	Cell Signaling Technology	4514	1: 250
Phosphor-MSK1-S^{376}	Rabbit	Abcam	ab32190	1: 250
Phospho-RB-$S^{807/811}$	Rabbit	Cell Signaling Technology	8516	1: 250
RB	Rabbit	Abcam	ab181616	1: 250
β-catenin	Rabbit	Cell Signaling Technology	8480	1: 250
Phospho-Stat3-Y^{705}	Rabbit	Cell Signaling Technology	9131	1: 250
Stat 3	Rabbit	Cell Signaling Technology	12640	1: 250
Phospho-Stat1-Y^{701}	Rabbit	Cell Signaling Technology	9167	1: 250
Phospho-Stat1-S^{727}	Rabbit	Cell Signaling Technology	8826	1: 250
Stat1	Rabbit	Cell Signaling Technology	14994	1: 250
Fibronectin	Rabbit	Abcam	ab45688	1: 250
RSK	Rabbit	Cell Signaling Technology	9355	1: 250
RBPSUH	Rabbit	Cell Signaling Technology	5313	1: 250
SMAD2	Rabbit	Cell Signaling Technology	5339	1: 250
Src	Rabbit	Cell Signaling Technology	2123	1: 250
NF-κB p65	Rabbit	Cell Signaling Technology	8242	1: 250

Notch1	Rabbit	Cell Signaling Technology	3608	1: 250
Notch2	Rabbit	Cell Signaling Technology	5732	1: 250
Jagged1	Rabbit	Cell Signaling Technology	2620	1: 250
Jagged2	Rabbit	Cell Signaling Technology	2210	1: 250
Phospho-Met-$T^{1234/1235}$	Rabbit	Cell Signaling Technology	3077	1: 250
Met	Rabbit	Cell Signaling Technology	8198	1: 250
Dvl2	Rabbit	Cell Signaling Technology	3224	1: 250
Dvl3	Rabbit	Cell Signaling Technology	3218	1: 250
CD133	Rabbit	Cell Signaling Technology	64326	1: 250
CHD1L	Rabbit	Cell Signaling Technology	13460	1: 250
Claudin1	Rabbit	Cell Signaling Technology	13255	1: 250
EpCAM	Rabbit	Proteintech	21050-1-AP	1: 250
Integrin β1	Rabbit	Cell Signaling Technology	9699	1: 250
TGFβ-R1	Rabbit	Abcam	ab31013	1: 250
Phospho-FAK-Y^{397}	Rabbit	Cell Signaling Technology	8556	1: 250
c-Myc	Rabbit	Abcam	Ab32072	1: 100
SOS1	Rabbit	Cell Signaling Technology	12409	1: 250
Transferrin Receptor	Mouse	Thermo Fisher Scientific	136800	1: 250
Phospho-c-Jun-S^{63}	Rabbit	Cell Signaling Technology	2361	1: 250
c-Jun	Rabbit	Cell Signaling Technology	9165	1: 250
Phospho-MAPKAPK-2 -T^{334}	Rabbit	Cell Signaling Technology	3007	1: 250
CyclinD1	Rabbit	Abcam	ab31013	1: 100

γ-Tubulin	Mouse	Sigma-Aldrich	T5326	1: 1000
