# Supplementary material for: Proteolytic EphA2 fragments cooperatively promote hepatocellular carcinoma progression
Source: Cell Death Dis. 2026 Jun 29;17(1):799. doi: 10.1038/s41419-026-09024-1 (PMC13575192; doi:10.1038/s41419-026-09024-1)
Supplement: Supplementary file 3 [file 41419_2026_9024_MOESM3_ESM.pdf]

## Supplementary Materials and Methods

### Antibodies, inhibitors and activator

Anti-EphA2 mouse monoclonal antibody (mAb; C-3) and anti-Ephrin-A1 rabbit polyclonal antibody (V-18; pAb) were purchased from Santa Cruz (St Louis, MO). Anti-phosphorylated AKT-$S^{473}$ rabbit (D9E), anti-AKT mouse (40D4), anti-phosphorylated GSK3β-$S^{9}$ rabbit (D85E12), anti-GSK3β rabbit (D5C5Z), anti-phosphorylated p38MAPK-$T^{180}$/$Y^{182}$ rabbit (D3F9), anti-phosphorylated ERK1/2-$T^{202}$/$Y^{204}$ mouse (D13.14.4E) anti-phosphorylated MEK1/2-$S^{217/221}$ rabbit (41G9), anti-phosphorylated SEK1/MKK4-$S^{257}$ rabbit (C36C11), anti-phosphorylated EphA2-$Y^{588}$ rabbit (D7X2L) and anti-phosphorylated EphA2-$S^{897}$ rabbit (D9A1) mAbs were purchased from Cell Signaling Technology (Danvers, MA, USA). Anti-Flag mouse (FLA-1), anti-HA mouse (TANA2), anti Myc mouse (My3) mAbs were purchased from Medical & Biological Laboratories (MBL, Japan). Anti-Transferrin Receptor mouse (H68.4) mAb was purchased from Thermo Fisher Scientific (Waltham, MA, USA). Anti-MT1-MMP mouse (222-1D8) mAb was made in our laboratory. Anti-β-tubulin mouse mAb (E7) was purchased from Developmental Studies Hybridoma Bank (IowaCity, IA). EGFR tyrosine kinase inhibitor Gefitinib and GSK3β activator Sodium nitroprusside (SNP)(1,2) were purchased from Sigma-Aldrich (St. Louis, MO). AKT inhibitor MK2206 and MEK inhibitor Binimetinib were purchased from Cayman Chemical (Ann Arbor, MI). MKK4 inhibitor Darizmetinib was purchased from TargetMol (Boston, MA).

### Purification of the recombinant EphrinA1-Fc (EA1) and EphA2-NF-Flag

MDCK cells stably expressing EA1 and Expi 293 cells stably expressing EphA2-NF-FLAG were generated using the ViraPower Lentiviral Expression System (Thermo Fisher Scientific) according to the manufacturer's instructions. Conditioned medium (CM) was collected from cells and purified using the MAbTrap™ Kit (Cytiva, Freiburg, Germany) or DDDDK-tagged Protein

PURIFICATION GEL (MBL). The affinity-purified proteins were dialyzed against ice-cold PBS and concentrated using Amicon Ultra 10K (Millipore). In proliferation assays and signal transduction analyses, EA1 was used at a concentration of 1 μg/ml, and EphA2-NF-Flag was used at a concentration of 5 μg/ml.

**Biotinylation of cell surface proteins**

SK-Hep1 and JHH6 were seeded on 10 cm dish and grown to confluence, then washed with ice-cold PBS. The cell was incubated with 0.3 mg/ml sulfo-NHS-Lc-biotin (Pierce) in PBS for 2 hrs on ice. After incubation, biotin reaction was quenched with 0.1 M glycine and then lysed using TNET-S buffer (20 mM Tris-HCl (pH 7.5), 0.15 M NaCl, 1 mM EDTA, 1 mM DTT, and 1% NP40) and a protease inhibitor cocktail (Millipore). The biotinylated membrane proteins were collected using streptavidin beads, washed with 1% CHAPS and then added with 2x sample buffer (100 mM Tris-HCl (pH 6.8), 4% SDS, 10% 2-mercaptoethanol, 0.2% bromophenol blue, and 20% glycerol) to prepare samples for Western blotting.

**Protein extraction and Western blotting**

Hep3B cells were lysed using TBT lysis buffer (50 mM Tris-HCl (pH 7.5), 0. 15 M NaCl, 1% TritonX-100, and 0.005% Briji35) containing protease inhibitor cocktail (Millipore, Billerica, MA) and PhosSTOP (Sigma-Aldrich). The lysates were transferred to polyvinylidene fluoride membranes after SDS-PAGE under reducing conditions. After blocking with 5% nonfat dry milk or 2% bovine serum albumin (BSA), membranes were incubated with primary antibodies at 4℃ overnight at the indicated concentration (Table S1). The membranes underwent another incubation with horseradish peroxidase labeled secondary antibodies for 1 h at room temperature. The bands were detected using chemiluminescence using EzWestLumi plus (ATTO) and visualized using

Chemidoc Imaging System (Bio-Rad, Hercules,CA).

**Immunofluorescence microscopy.**

Coverslips were placed in a 24-well plate and coated with poly-L-lysine to allow cell adhesion. Hep3B cells were then seeded at 5,000 cells/well and incubated overnight. The cells were then washed with PBS and incubated 4% paraformaldehyde in PBS for 10 min at room temperature and permeabilized with 0.01% Triton X-100 in PBS for 10 min at room temperature. Cells were then washed three times in PBS and blocked for 1 h with 5% goat serum in PBS. Cells were incubated with primary antibodies with 5% goat serum in PBS at 4 °C overnight, washed three times with PBS, and incubated with secondary antibodies (anti mouse Alexa Fluor 488 or anti rabbit Alexa Fluor 488) with 5% goat serum in PBS for 1h at room temperature. After three washes with PBS, Transfer the immunostained coverslips to a glass slide. At this point, coverslips were mounted using VECTASHIELD Mounting Medium containing 4', 6-diaminido-2-phenylindole (DAPI) (Vector Laboratories, Burlingame, CA). Images were taken using fluorescence microscope (Ti2-E, Nikon)

**Immunoprecipitation**

Recombinant EphrinA1-Fc and EphA2-NF-Flag were incubated overnight at 4℃ with anti-FLAG mAb in PBS. rProtein-A Sepharose Fast Flow (GE Healthcare) was added and incubated at room temperature for 2h. The Sepharose beads were washed, dissolved in 2x sample buffer and then subjected to Western blotting.

**Reverse-phase protein array (RPPA)**

RPPA assay was performed as previously described (3-5). Details of the antibodies used are shown in Table S2. In detail, Hep3B cells were lysed using T-PER protein extraction reagent (Thermo Fisher Scientific) supplemented with inhibitors (100 mM NaF, 1mM $Na_3VO_4$, 10 mM $Na_2H_2P_2O_7$, 1 mM EDTA, PhosSTOP (Sigma-Aldrich) and protease inhibitor cocktail (Millipore). The lysates were collected by scraper from the culture dishes and centrifuged at 14,000 rpm for 20 min at 4 °C. The supernatants were used as a clear lysate. Protein concentrations were measured by a Bradford protein assay (Bio-Rad) Lysates were boiled in 2% SDS and 2.5% 2-mercaptoethanol and adjusted to approximately 2.0 mg/mL. The lysates were printed in four replicates onto nitrocellulose-coated slides (Grace Bio-Labs, Bend, OR, USA) using an Aushon Biosystems 2470 Arrayer (Burlington, MA, USA). After blocking with a blocking buffer containg 0.1% Tween-20 (LI-COR Biosciences, Lincoln, NE, USA), the blotted slides were probed with validated primary antibodies at the indicated concentration (Table 2), followed by secondary antibodies conjugated to the infrared dyes IRDye 680RD and 800CW (LI-COR Biosciences). Slides were scanned using an ODYSSEY scanner (LI-COR Biosciences). The signal intensity of each spot was analyzed using Image Studio (LI-COR Biosciences) according to the manufacturer's instructions. For each slide, RPPA data of protein expression and phosphorylation for each cell line were normalized by the RPPA intensity of the housekeeping protein tubulin, log2 transformed, and Z-score standardized across Hep3B cells treated under each condition.

**References**

1. Mariappan MM, Prasad S, D'Silva K, Cedillo E, Sataranatarajan K, Barnes JL*, et al.* Activation of glycogen synthase kinase 3beta ameliorates diabetes-induced kidney injury. J Biol Chem **2014**;289:35363-75

2. Jiang X, Zhang W, Xie S. METTL3 inhibits microglial pyroptosis in neonatal hypoxia-ischemia encephalopathy by regulating GPR39 expression in an m6A-HuR-dependent manner. Neuroscience **2024**;563:175-87

3. Suzuki M, Muroi A, Nojima M, Numata A, Takasaki H, Sakai R*, et al.* Utility of a Reverse Phase Protein Array to Evaluate Multiple Biomarkers in Diffuse Large B-Cell Lymphoma. Proteomics Clin Appl **2020**;14:e1900091

4. Osumi H, Muroi A, Sakahara M, Kawachi H, Okamoto T, Natsume Y*, et al.* Evaluation of the RAS signaling network in response to MEK inhibition using organoids derived from a familial adenomatous polyposis patient. Sci Rep **2020**;10:17455

5. Nakamura T, Nada S, Matsumoto M, Loling Othman N, Kosako H, Ikeda K*, et al.* Amino acid-dependent TSC2 dephosphorylation by lysosome-PP2A regulates mTORC1 signaling transduction. Life Sci Alliance **2025**;8

**Supplementary Material**

**Figure S1.** Detection of EphA2 and EphA2-CF in eight HCC cells using western blotting under long-exposure conditions (corresponding to Figure 1A).

**Figure S2.** IL-1β induces EphA2 expression, MT1-MMP activation, and EphA2 cleavage in HCC cells.

Western blot analysis showing time-dependent induction of EphA2 expression, MT1-MMP activation, and EphA2-CF production in SK-Hep1 cells treated with IL-1β for the indicated time points (0, 12, and 24 h). Tubulin was used as a loading control. These results suggest that inflammatory stimuli enhance MT1-MMP–mediated EphA2 cleavage.

**Figure S3.** Time-course images of spheroid formation in Hep3B cells.

Representative time-lapse images showing spheroid formation at 0, 1, 4, 8, and 12 days in mock cells and EphA2 (#1 and #2)- and EphA2-CF (#1 and #2)-expressing cells cultured without (A; corresponding to Figure 2G) and with EA1 (B; corresponding to Figure 2H). Multiple representative fields are shown to illustrate spheroid growth over time. Scale bar, 200 µm.

**Figure S4.** Time-course images of spheroid formation in Hep3B cells.

Representative time-lapse images showing spheroid formation at 0, 1, 4, 8, and 12 days in mock and EphA2-CF (#1 and #2)-expressing cells cultured with EA1 (corresponding to Figure 3E). Multiple representative fields are shown to illustrate spheroid growth over time. Scale bar, 200 µm.

**Figure S5.** Effect of MEK inhibitor, MKK4 inhibitor and GSK3β activator on down-stream signaling pathways. Mock, EphA2 #1, and EphA2-CF #1 expressing cells treated with Binimetinib (A), Darizmetinib (B), or SNP (C) by Western blotting.

**Figure S6.** Time-course images of spheroid formation in Hep3B cells.

Representative time-lapse images showing spheroid formation at 0, 1, 4, 8, and 12 days in mock cells and EphA2 #1- and EphA2-CF #1-expressing cells cultured with EA1 (corresponding to Figure

6C). Multiple representative fields are shown to illustrate spheroid growth over time. Scale bar, 200 µm.

**Figure S7.** (A) Detection of enforced GSK3β-S9A-Myc proteins in SK-Hep1 cells using western blotting with anti-GSK3β and anti-Myc mAbs. β-tubulin was used as an internal control. (B) Comparison of proliferation activity in mock and GSK3β-S9A (#1 and #2)-expressing SK-Hep1 cells in the presence of EA1. Cell counts were performed after incubation for 3, 5, and 7 days.

**Figure S8.** Correlation analysis between GSK3β-S$^{9}$, EphA2-S$^{897}$ and AKT-S$^{473}$ phosphorylation in human HCC samples (n=184) from the Cancer Proteome Atlas (TCPA) database.

Scatter plots showing correlations between phosphorylation levels of EphA2-S897, GSK3β-S9, and AKT-S473 in human hepatocellular carcinoma samples derived from the TCPA dataset. (A) Correlation between EphA2-S897 and GSK3β-S9 phosphorylation. (B) Correlation between GSK3β-S9 and AKT-S473 phosphorylation (C) Correlation between AKT-S473 and EphA2-S897 phosphorylation. Each dot represents an individual tumor sample. Pearson correlation coefficients and corresponding P-values are indicated in each panel.

**Figure S9.** Time-course images of spheroid formation in Hep3B cells.

Representative time-lapse images showing spheroid formation at 0, 1, 4, 8, and 12 days in mock cells and EphA2 (#1 and #2)- and EphA2-CF #1-expressing cells treated with/without purified EphA2-NF protein in the presence of EA1 (corresponding to Figure 7F) Multiple representative fields are shown to illustrate spheroid growth over time. Scale bar, 200 µm.
