# Supplementary material for: Proteolytic EphA2 fragments cooperatively promote hepatocellular carcinoma progression
Source: Cell Death Dis. 2026 Jun 29;17(1):799. doi: 10.1038/s41419-026-09024-1 (PMC13575192; doi:10.1038/s41419-026-09024-1)
Supplement: Supplementary file 4 [file 41419_2026_9024_MOESM4_ESM.pdf]

Figure S1

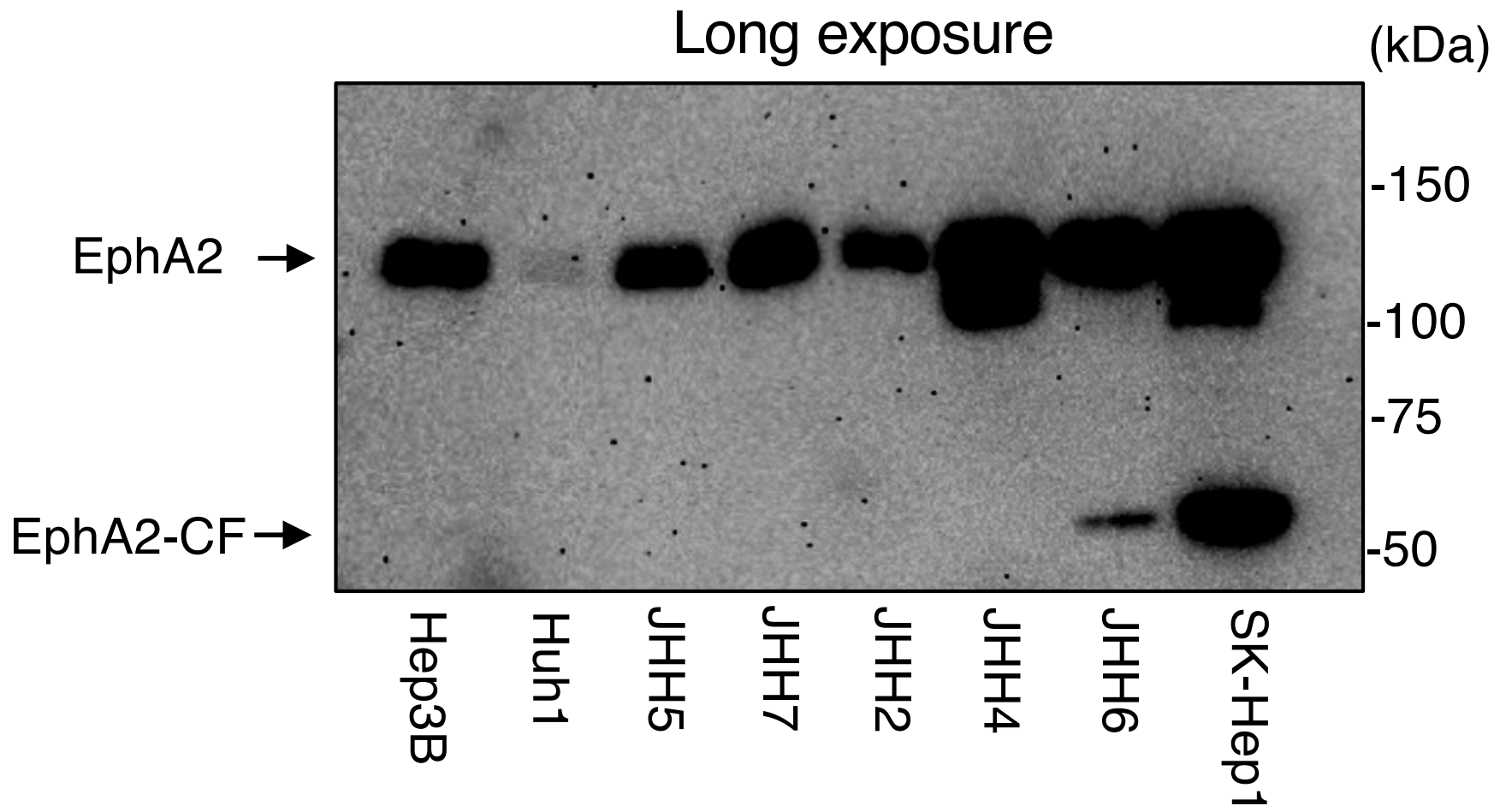

Long exposure
(kDa)
EphA2
EphA2-CF
-150
-100
-75
-50
Hep3B
Huh1
JHH5
JHH7
JHH2
JHH4
JHH6
SK-Hep1

Figure S2

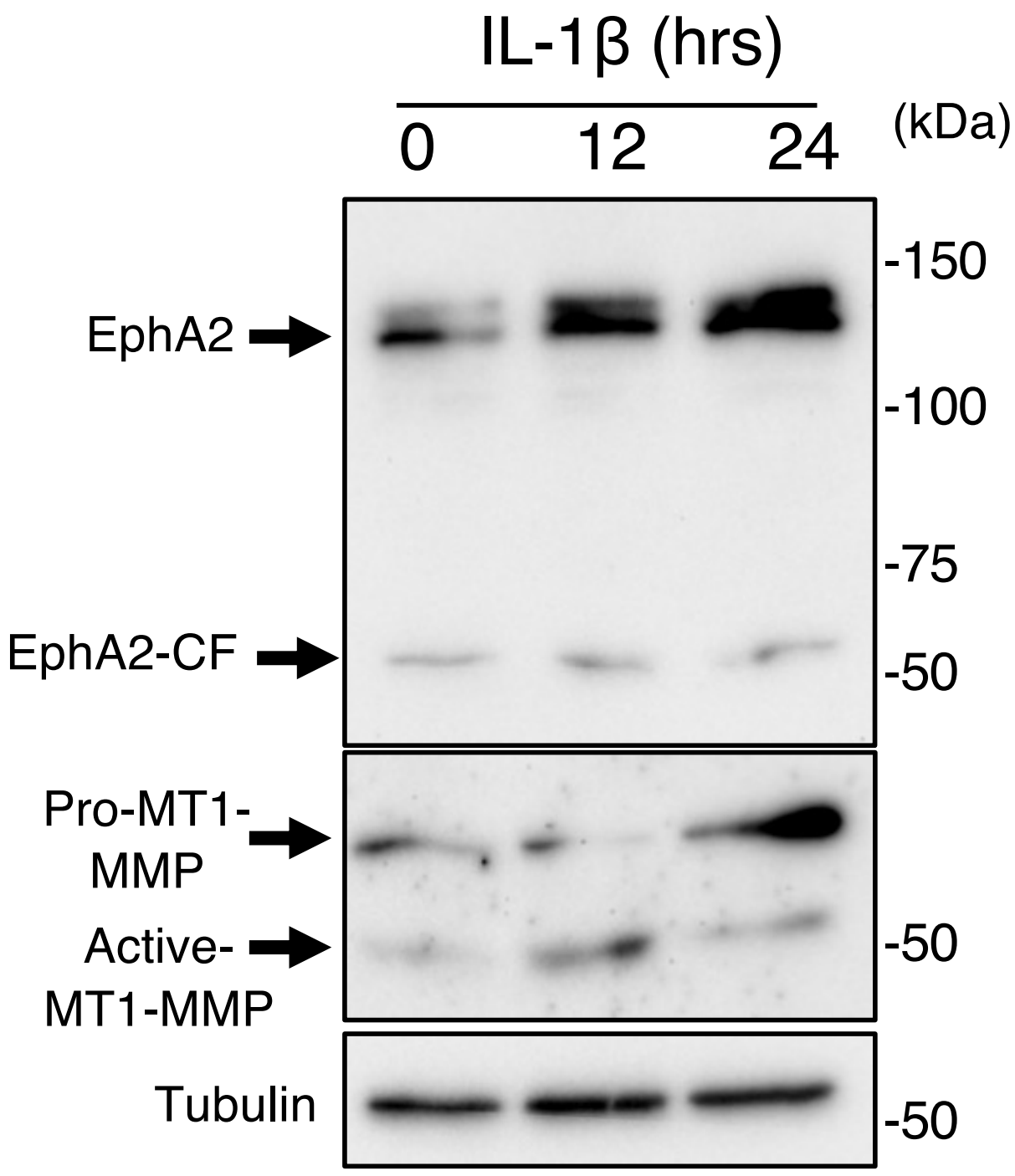
IL-1β (hrs)
0
12
24
(kDa)
EphA2
EphA2-CF
Pro-MT1-MMP
Active-MT1-MMP
Tubulin
-150
-100
-75
-50
-50
-50

Figure S3

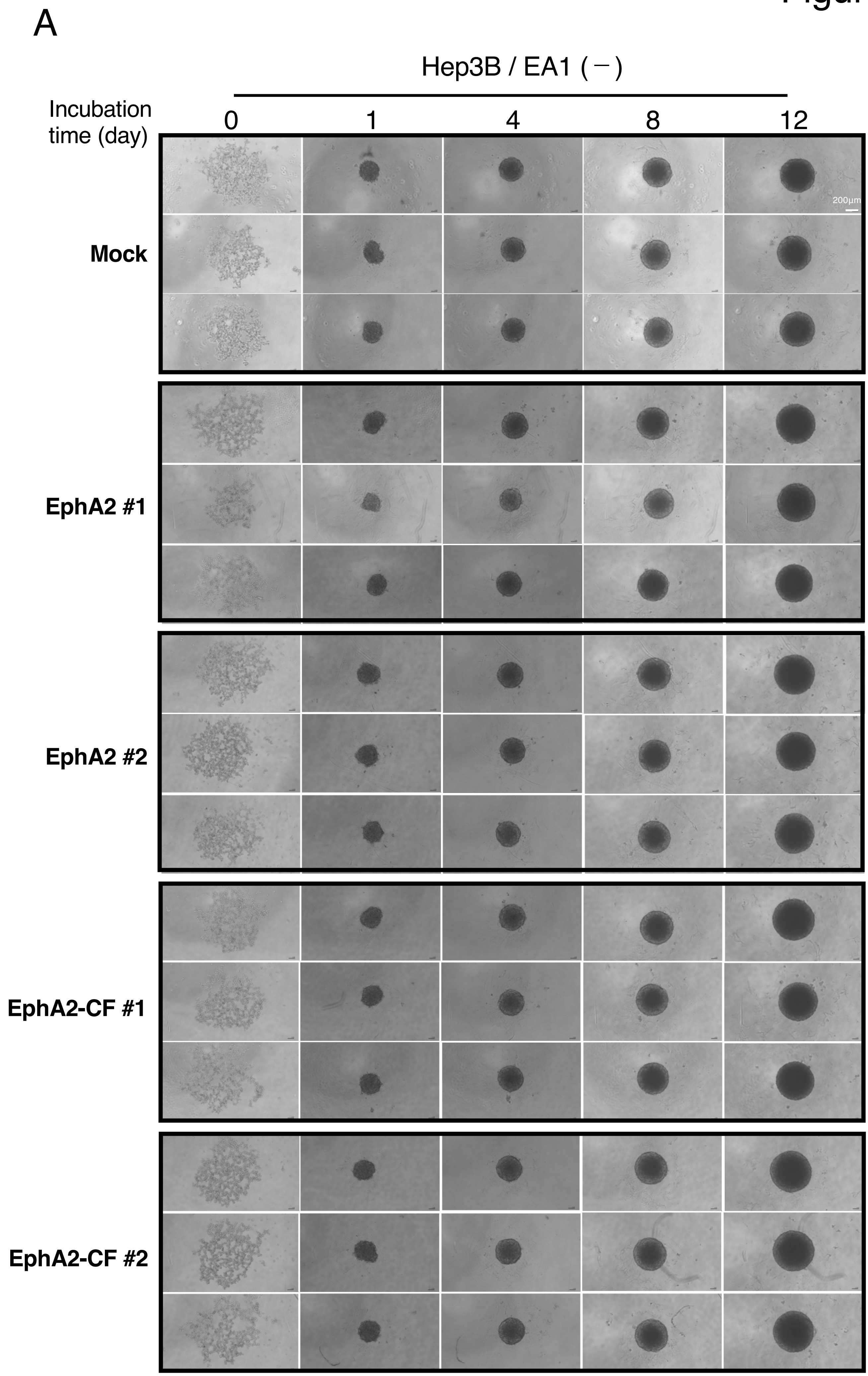
A
Hep3B / EA1 (−)
Incubation time (day)
0
1
4
8
12
200μm
Mock
EphA2 #1
EphA2 #2
EphA2-CF #1
EphA2-CF #2

Figure S3

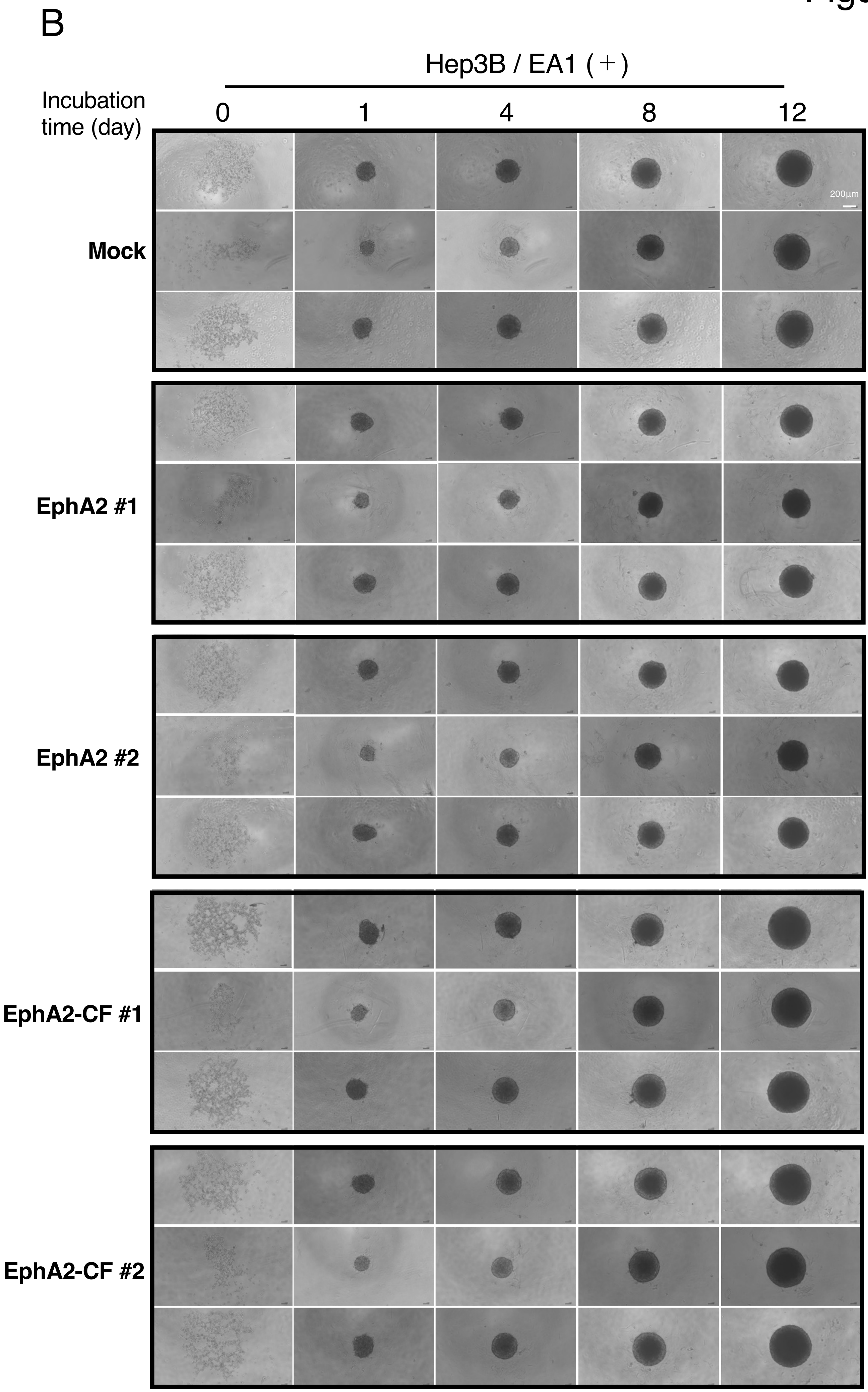
B
Hep3B / EA1 (+)
Incubation time (day)
0
1
4
8
12
200μm
Mock
EphA2 #1
EphA2 #2
EphA2-CF #1
EphA2-CF #2

Figure S4

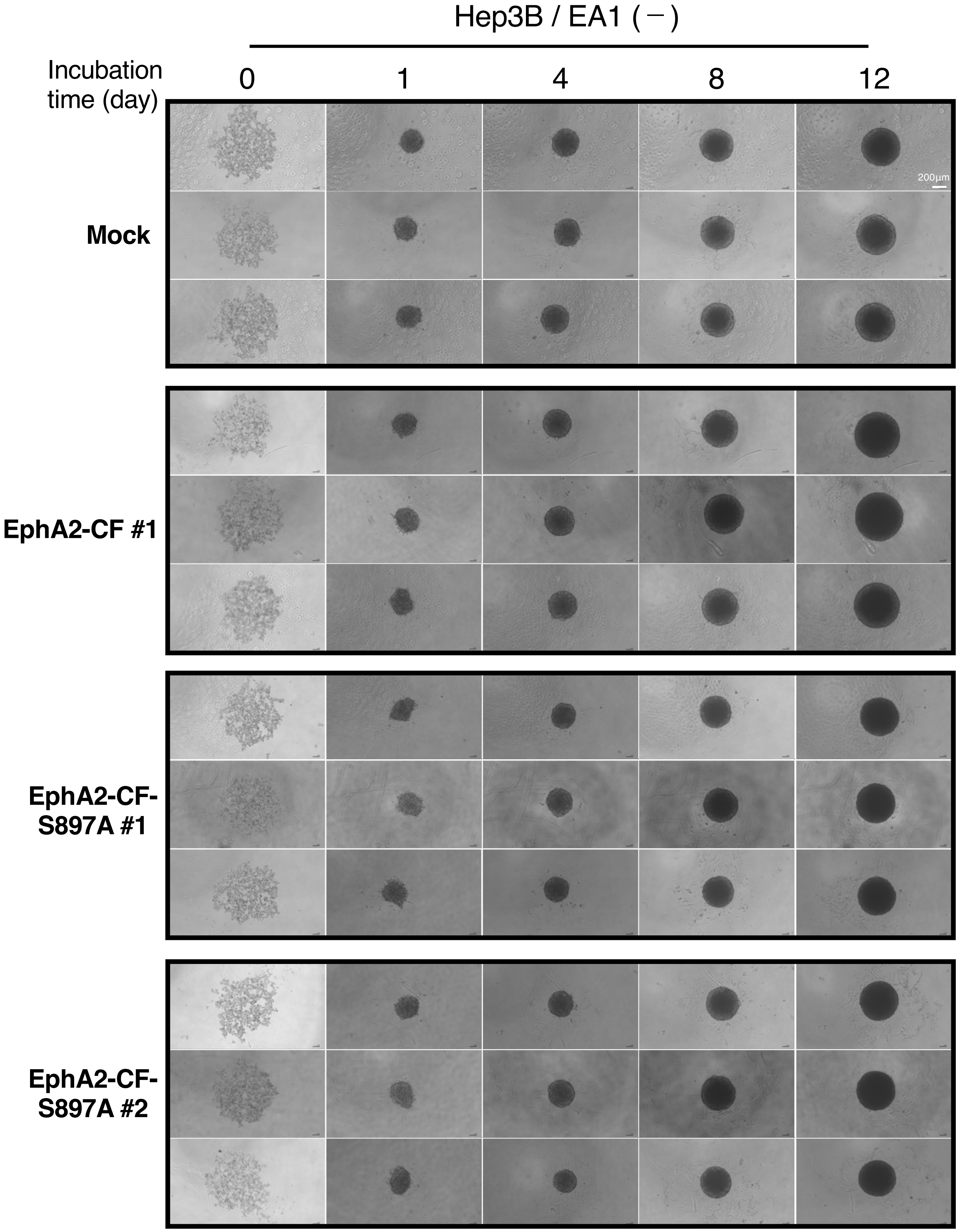

Hep3B / EA1 (－)
Incubation time (day)
0
1
4
8
12
200μm
Mock
EphA2-CF #1
EphA2-CF-S897A #1
EphA2-CF-S897A #2

Figure S5

A

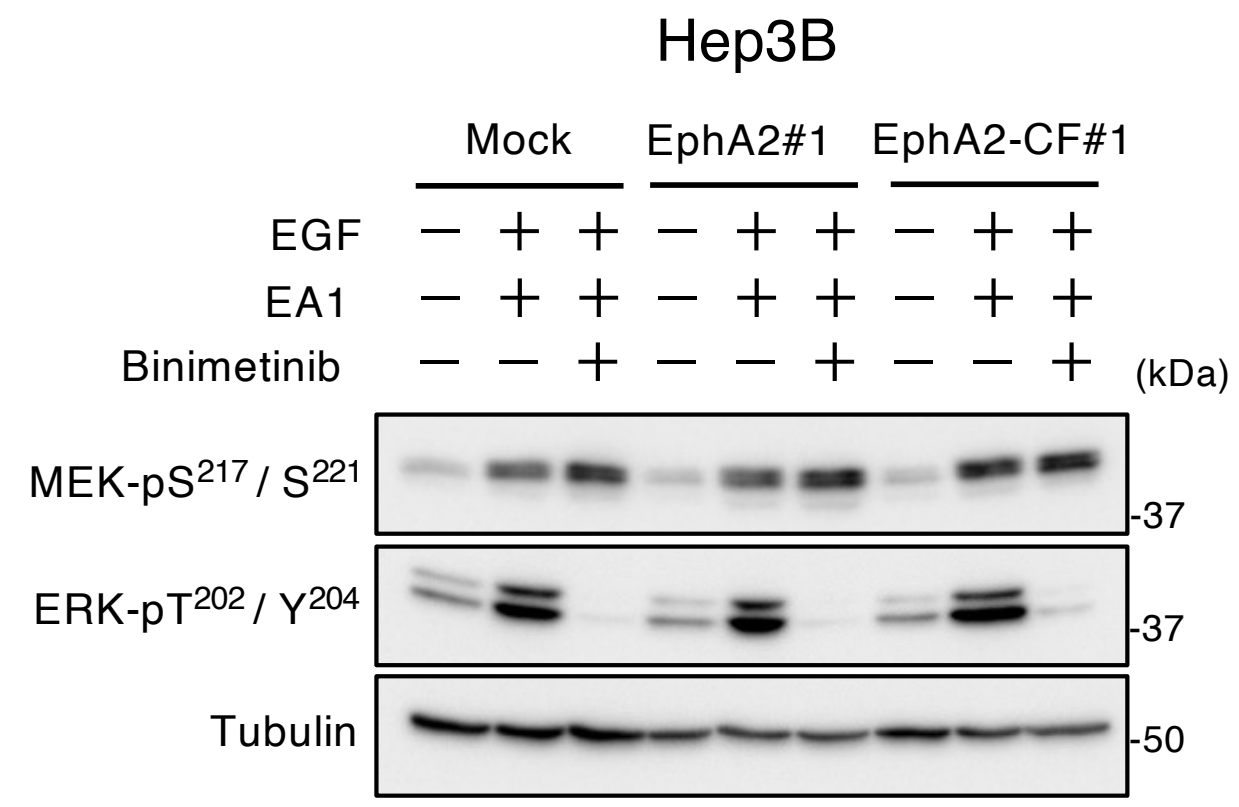

B

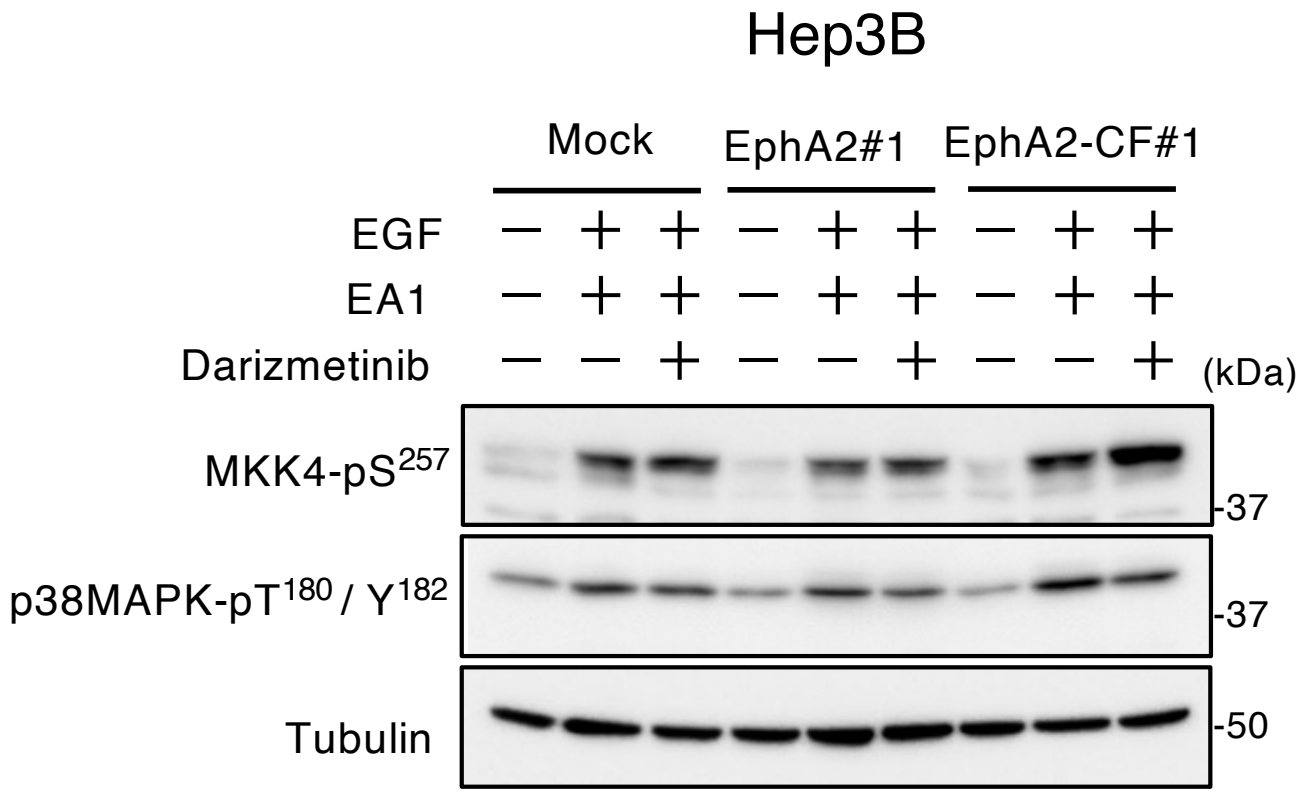

C

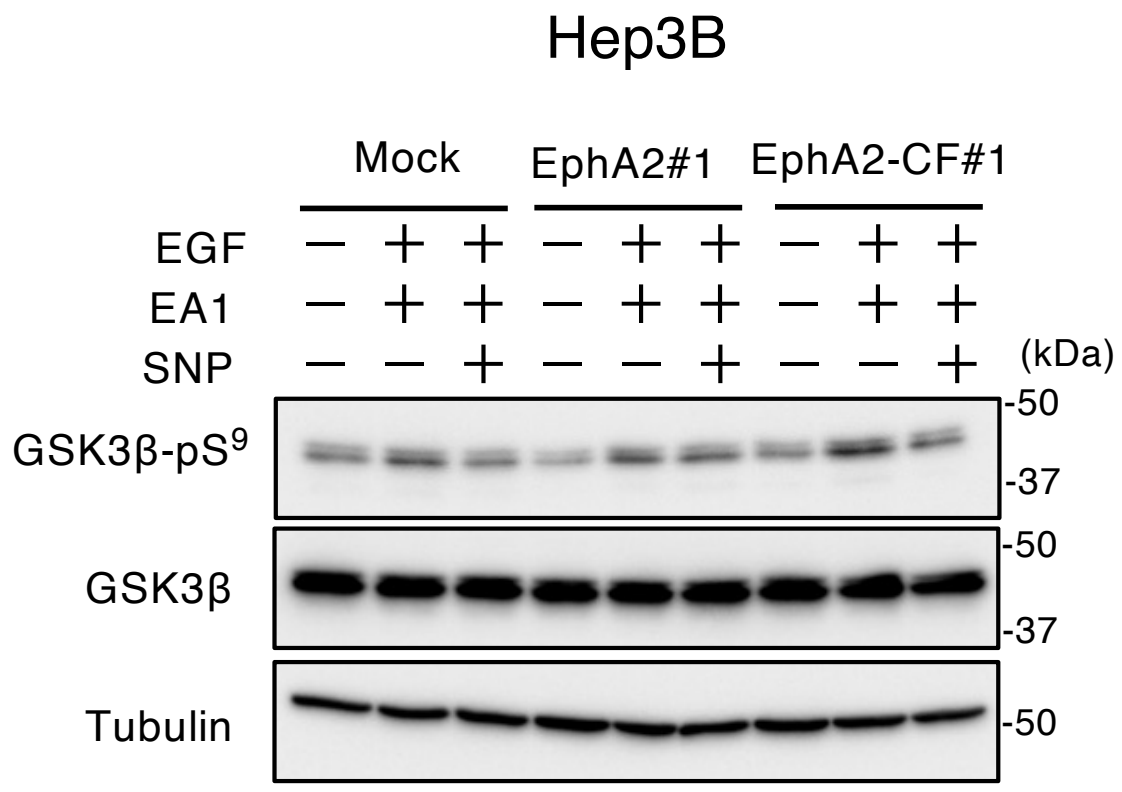

Figure S6

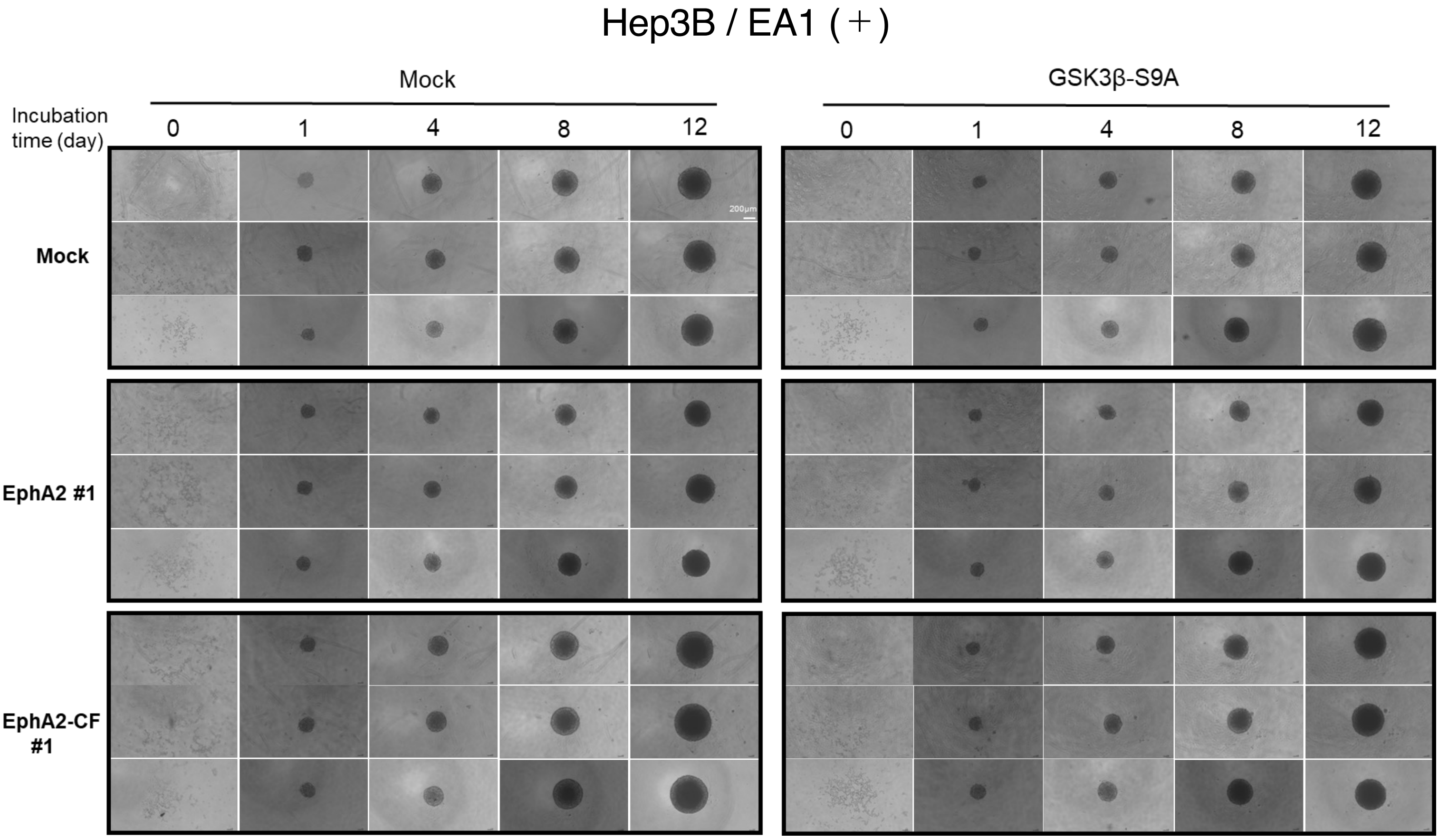
Hep3B / EA1 (+)
Mock
GSK3β-S9A
Incubation time (day)
0
1
4
8
12
Mock
EphA2 #1
EphA2-CF #1
200μm

Figure S7

A

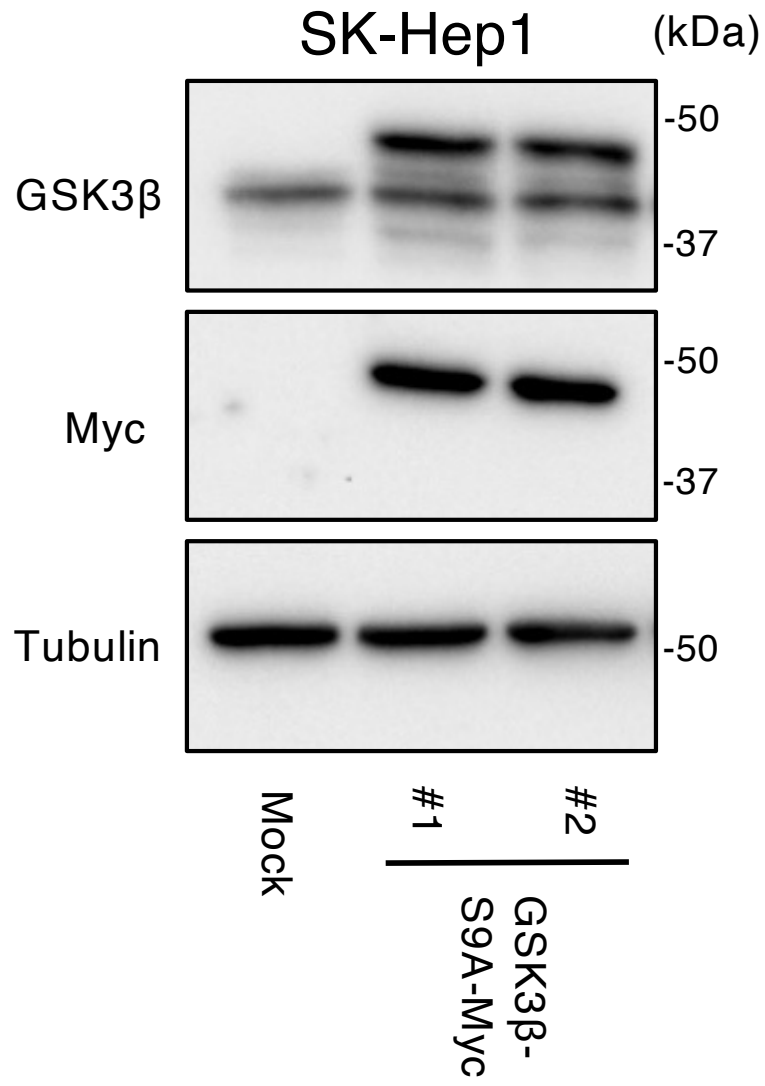
SK-Hep1
(kDa)
GSK3β
Myc
Tubulin
-50
-37
-50
-37
-50
Mock
#1
#2
GSK3β-S9A-Myc

B

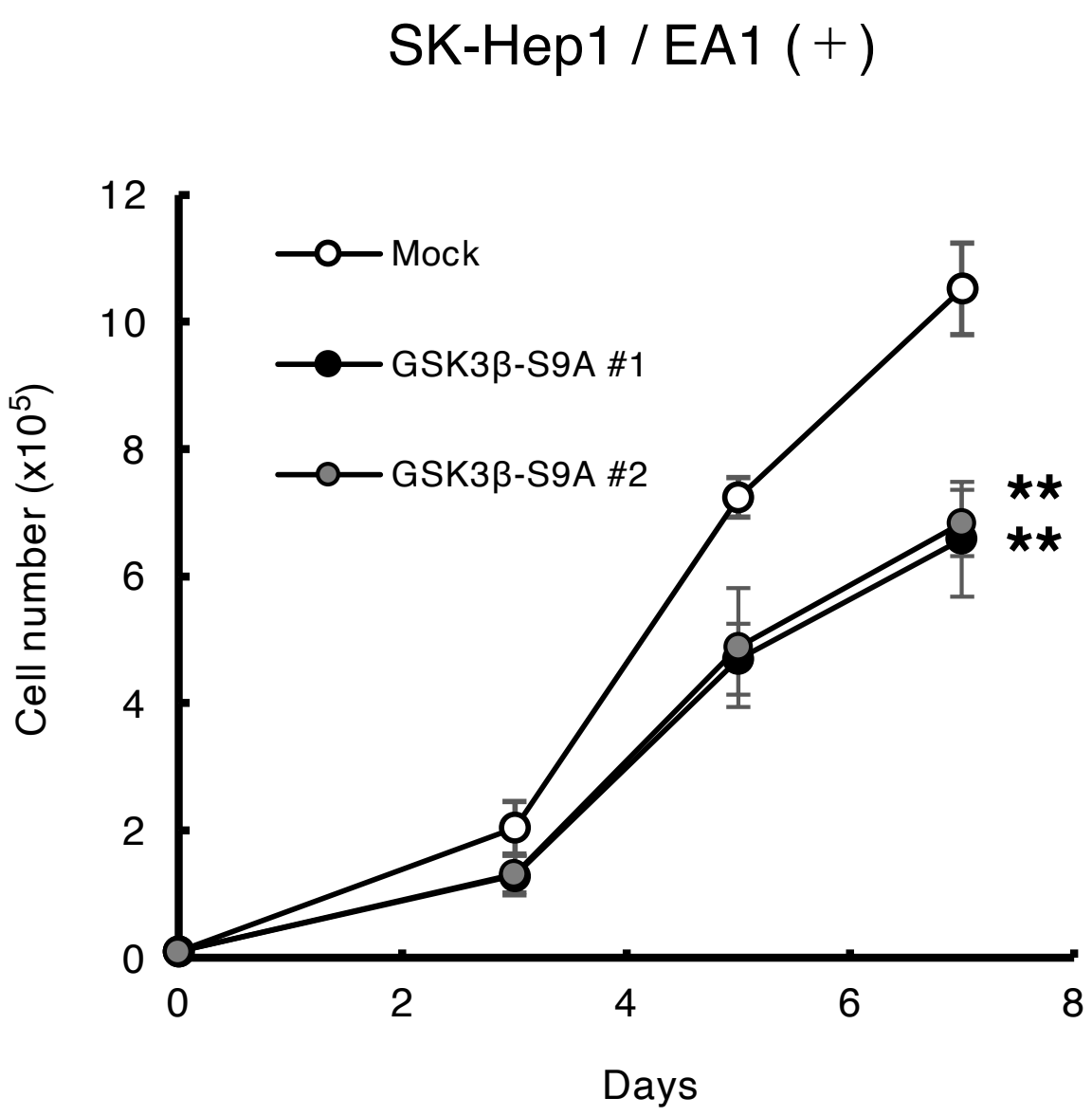
SK-Hep1 / EA1 (＋)
Mock
GSK3β-S9A #1
GSK3β-S9A #2
Cell number (x10^5)
Days
0
2
4
6
8
10
12
**
**

Figure S8

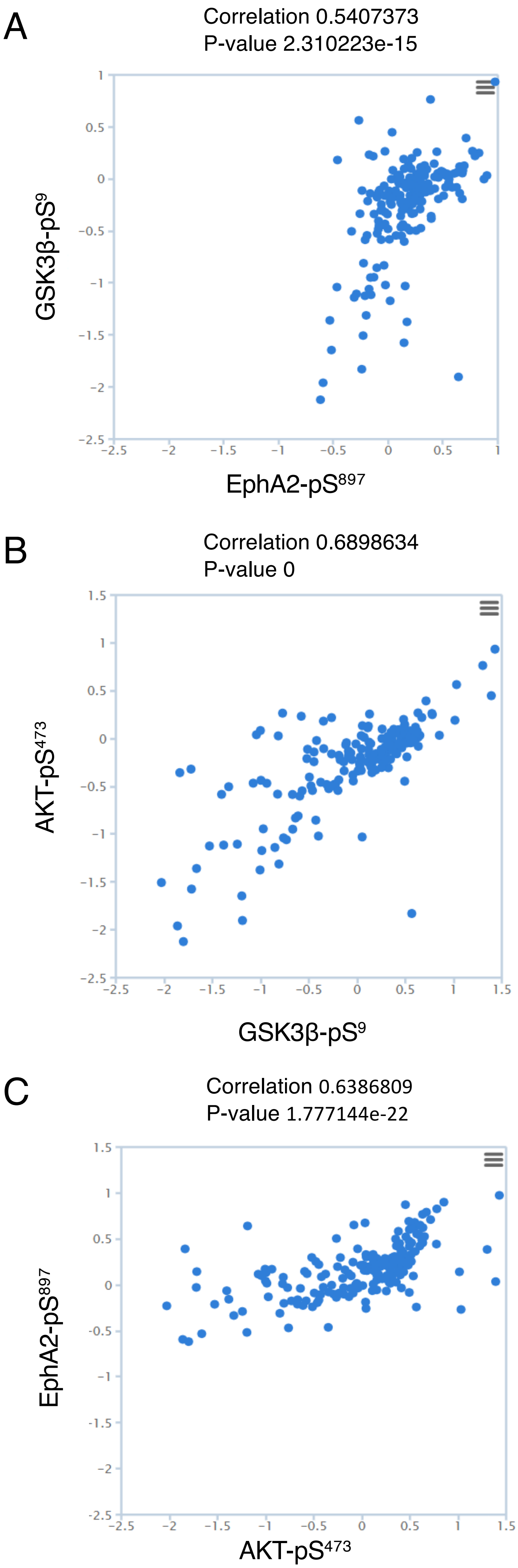
A
Correlation 0.5407373
P-value 2.310223e-15
GSK3β-pS9
EphA2-pS897
B
Correlation 0.6898634
P-value 0
AKT-pS473
GSK3β-pS9
C
Correlation 0.6386809
P-value 1.777144e-22
EphA2-pS897
AKT-pS473

Figure S9

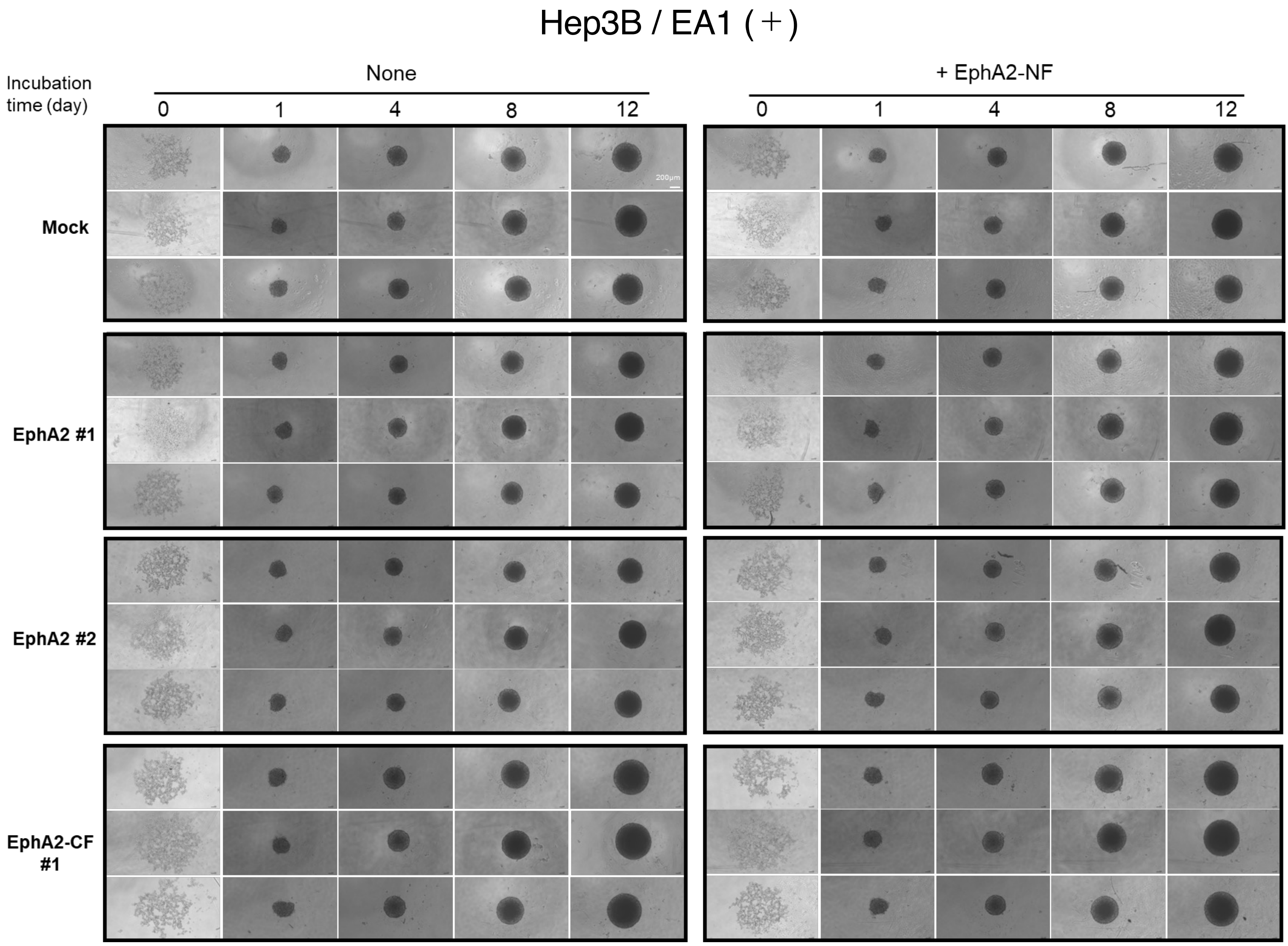
Hep3B / EA1 (＋)
Incubation time (day)
None
+ EphA2-NF
0
1
4
8
12
0
1
4
8
12
200μm
Mock
EphA2 #1
EphA2 #2
EphA2-CF #1
