# Supplementary material for: Proteolytic EphA2 fragments cooperatively promote hepatocellular carcinoma progression
Source: Cell Death Dis. 2026 Jun 29;17(1):799. doi: 10.1038/s41419-026-09024-1 (PMC13575192; doi:10.1038/s41419-026-09024-1)

Fig1A

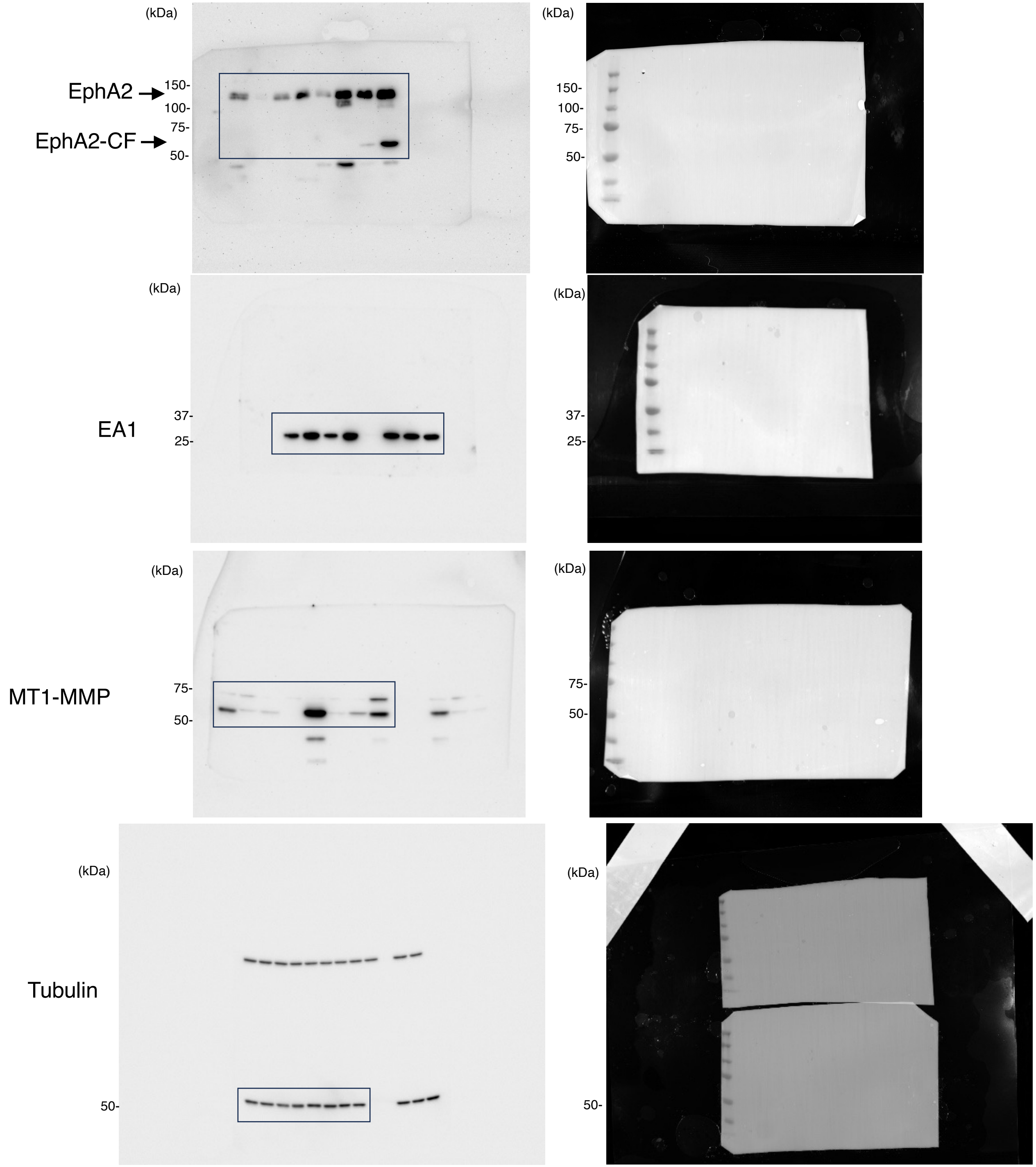
(kDa)
EphA2
EphA2-CF
150-
100-
75-
50-
(kDa)
150-
100-
75-
50-
(kDa)
EA1
37-
25-
(kDa)
37-
25-
(kDa)
MT1-MMP
75-
50-
(kDa)
75-
50-
(kDa)
Tubulin
50-
(kDa)
50-

Fig1B

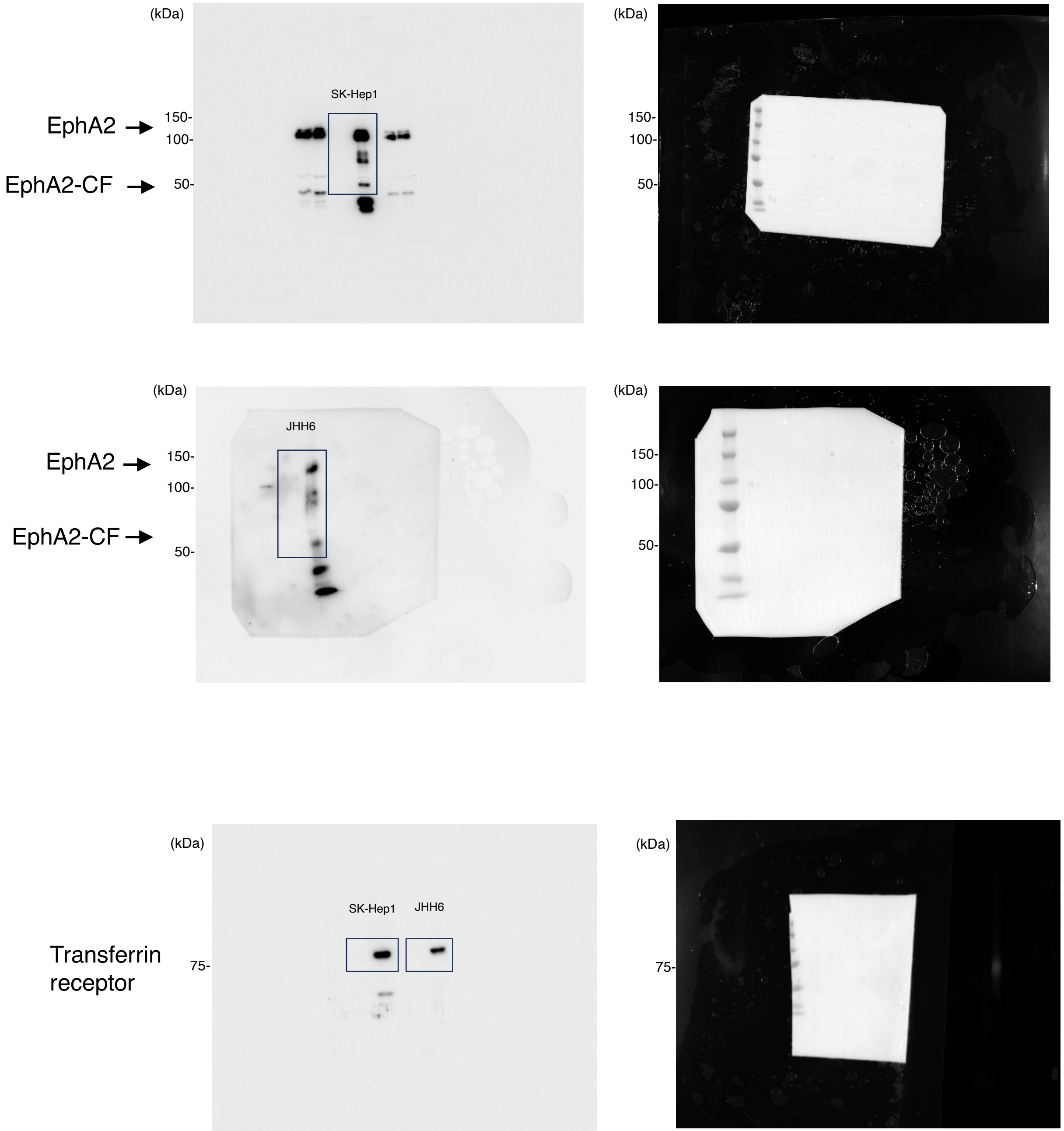

(kDa)
SK-Hep1
EphA2
EphA2-CF
150-
100-
50-
(kDa)
150-
100-
50-
(kDa)
JHH6
EphA2
EphA2-CF
150-
100-
50-
(kDa)
150-
100-
50-
(kDa)
SK-Hep1
JHH6
Transferrin receptor
75-
(kDa)
75-

Fig1C

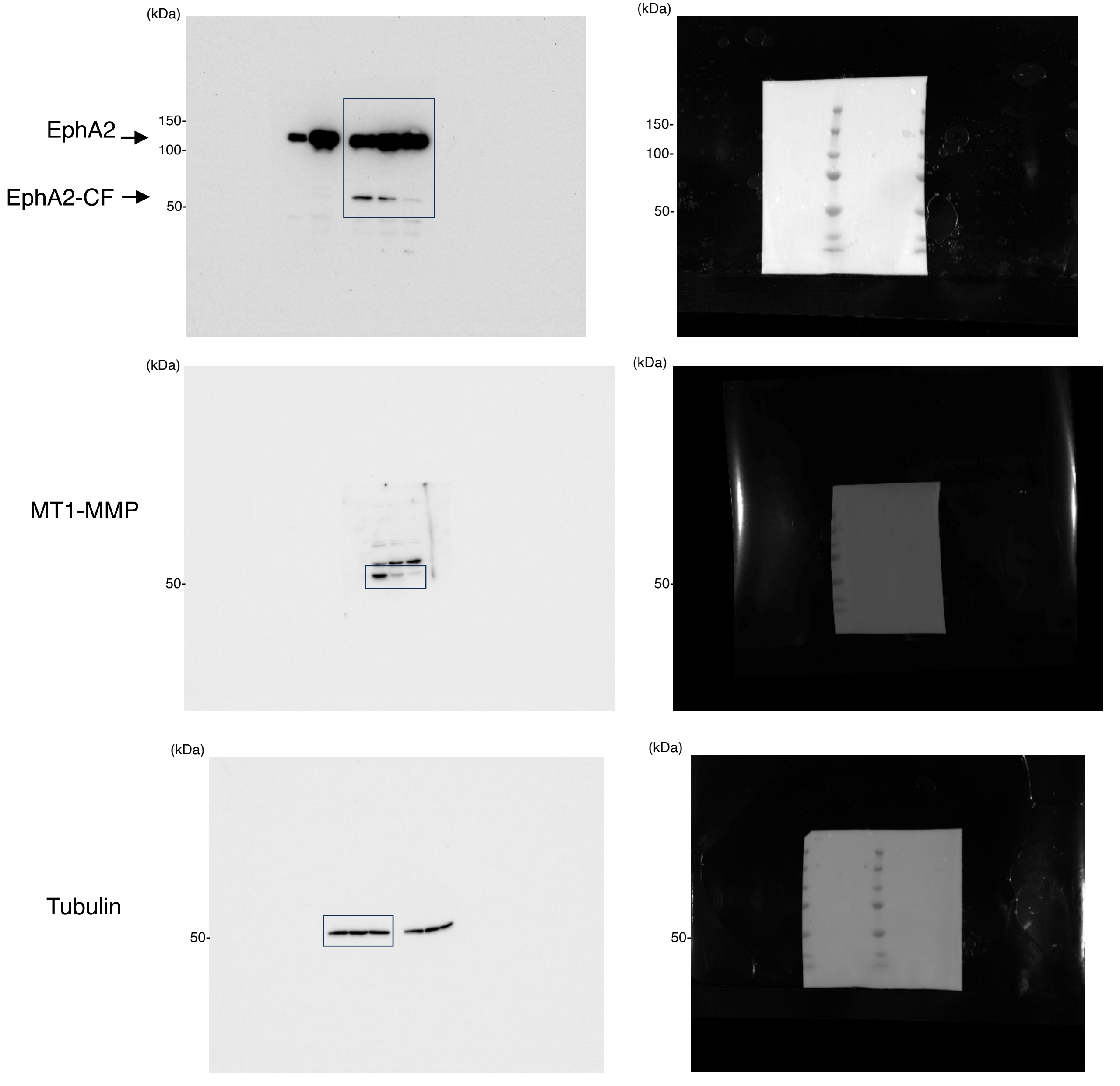
(kDa)
EphA2
150-
100-
EphA2-CF
50-
(kDa)
150-
100-
50-
(kDa)
MT1-MMP
50-
(kDa)
50-
(kDa)
Tubulin
50-
(kDa)
50-

Fig2A

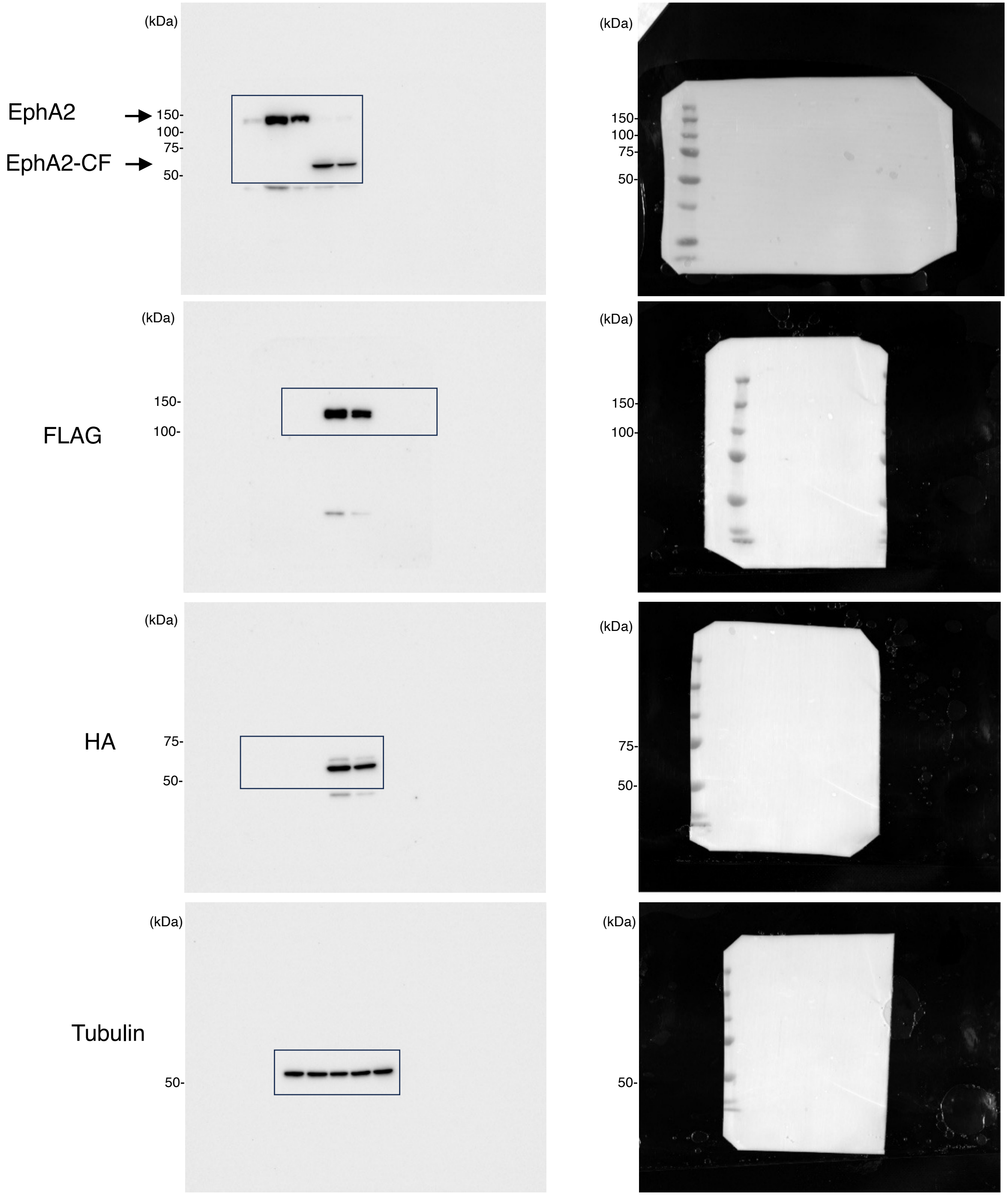

Fig3A

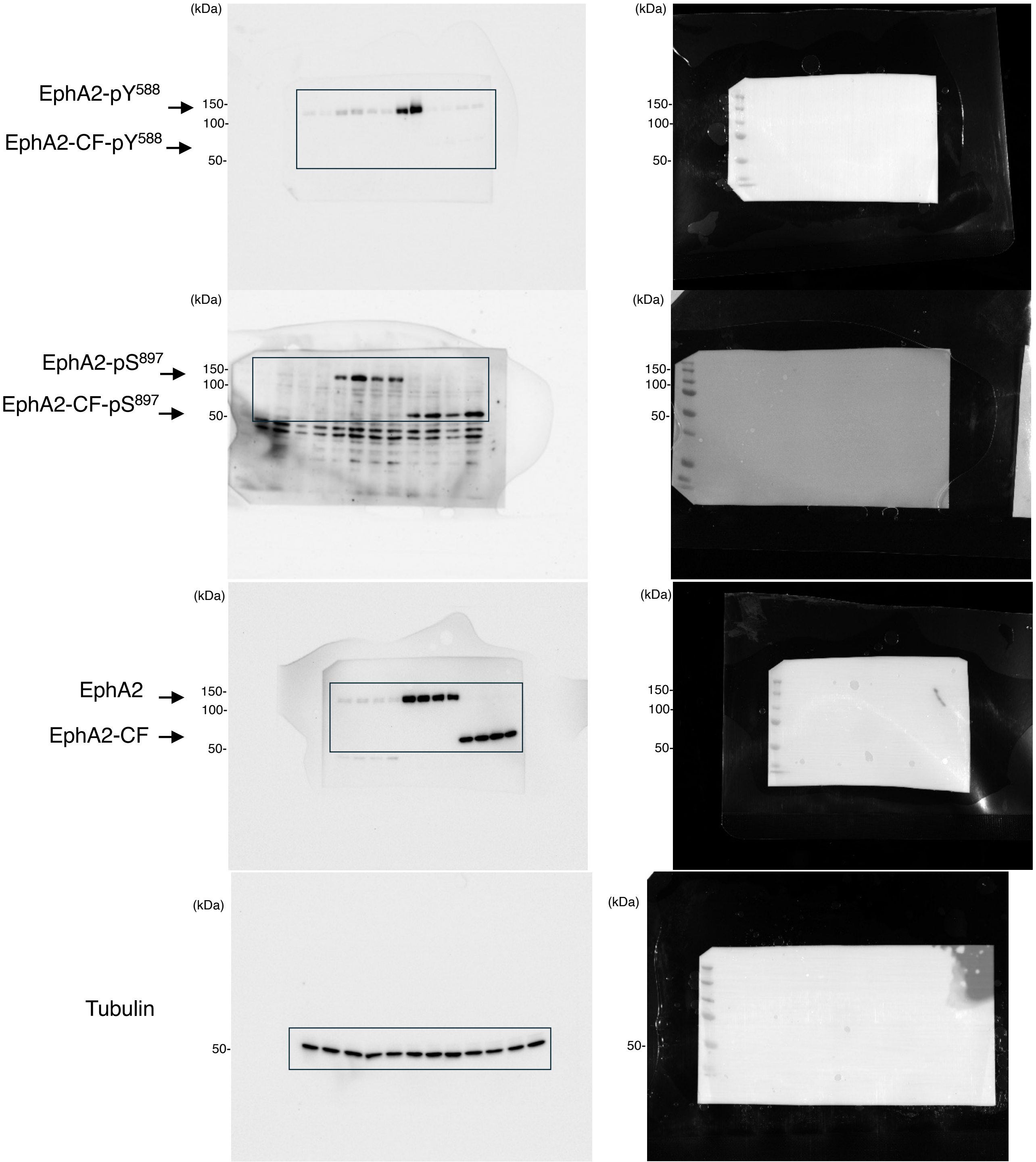

(kDa)
EphA2-pY588
150-
100-
EphA2-CF-pY588
50-
(kDa)
EphA2-pS897
150-
100-
EphA2-CF-pS897
50-
(kDa)
EphA2
150-
100-
EphA2-CF
50-
(kDa)
Tubulin
50-

Fig3B

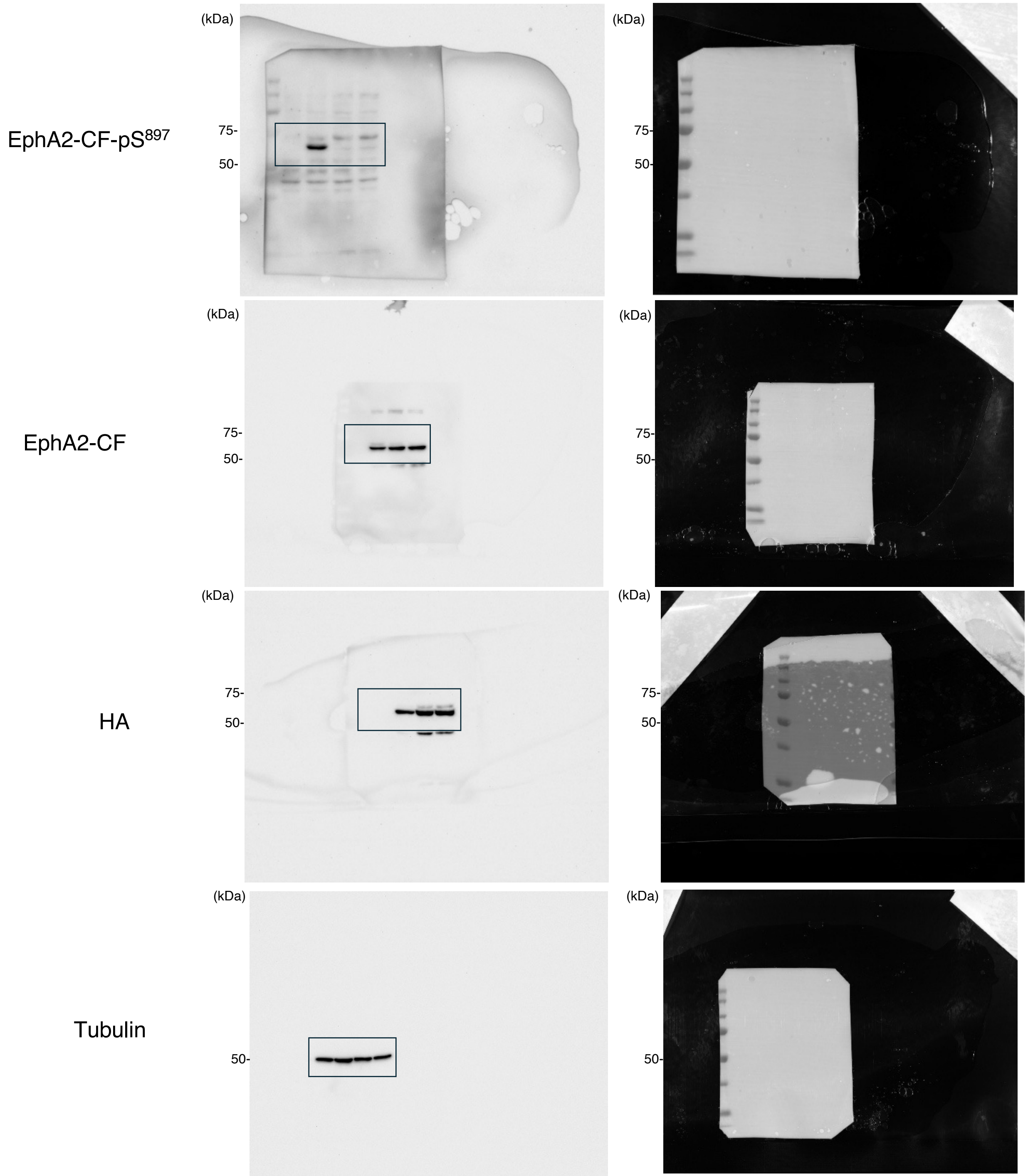

Fig6A

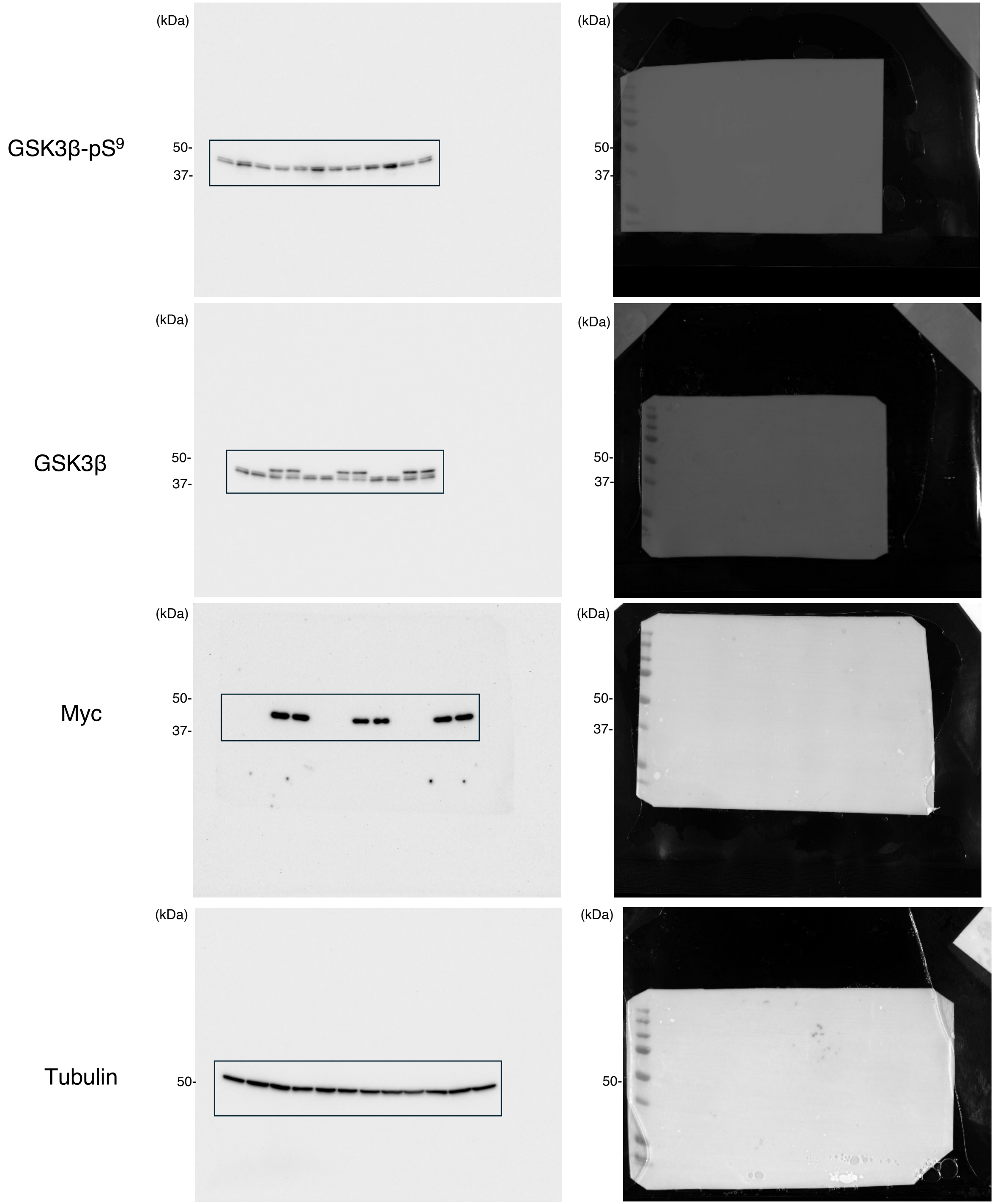

Fig6D

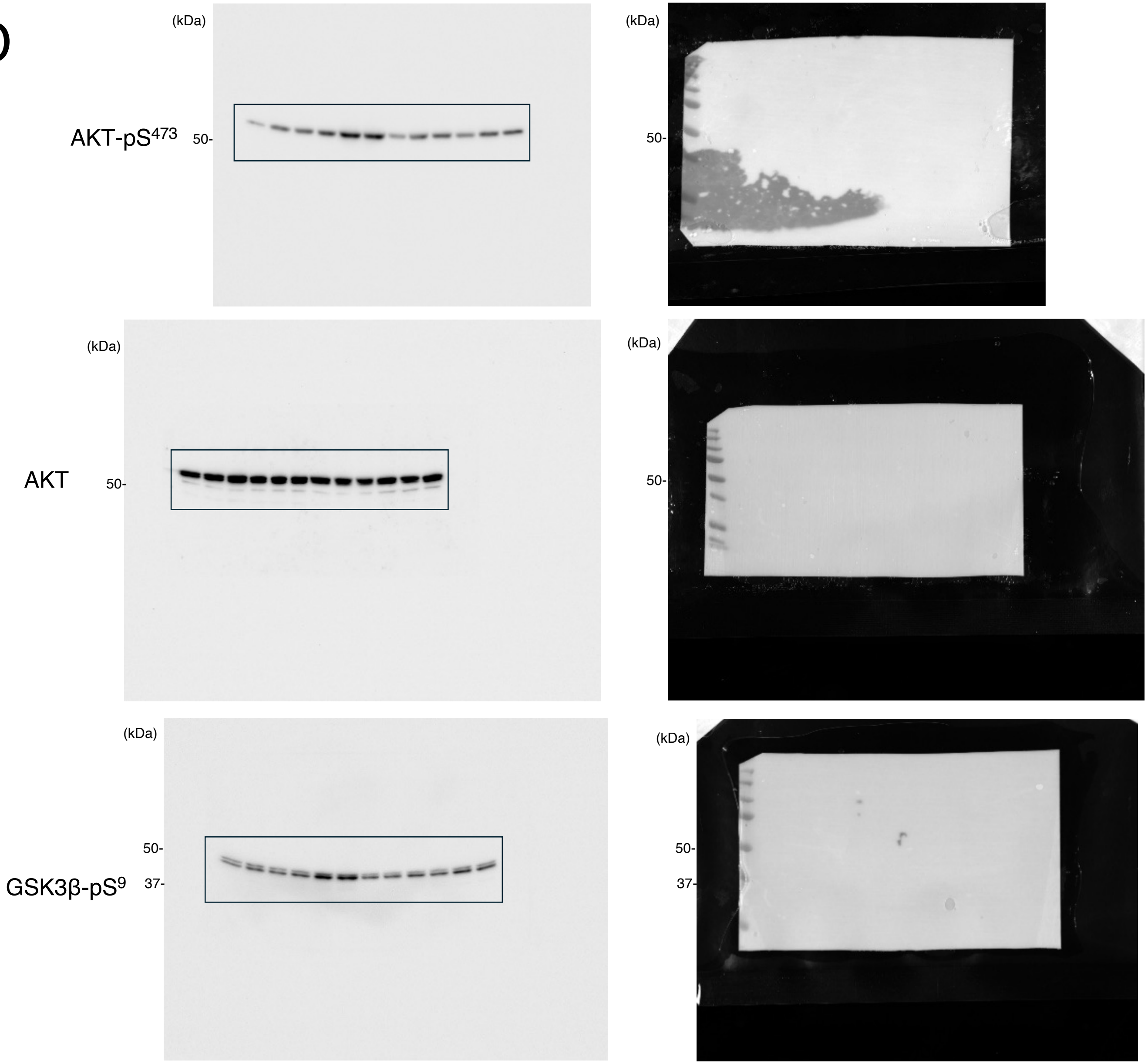
(kDa)
AKT-pS473
50-
(kDa)
50-
(kDa)
AKT
50-
(kDa)
50-
(kDa)
50-
GSK3β-pS9
37-
(kDa)
50-
37-

Fig6D

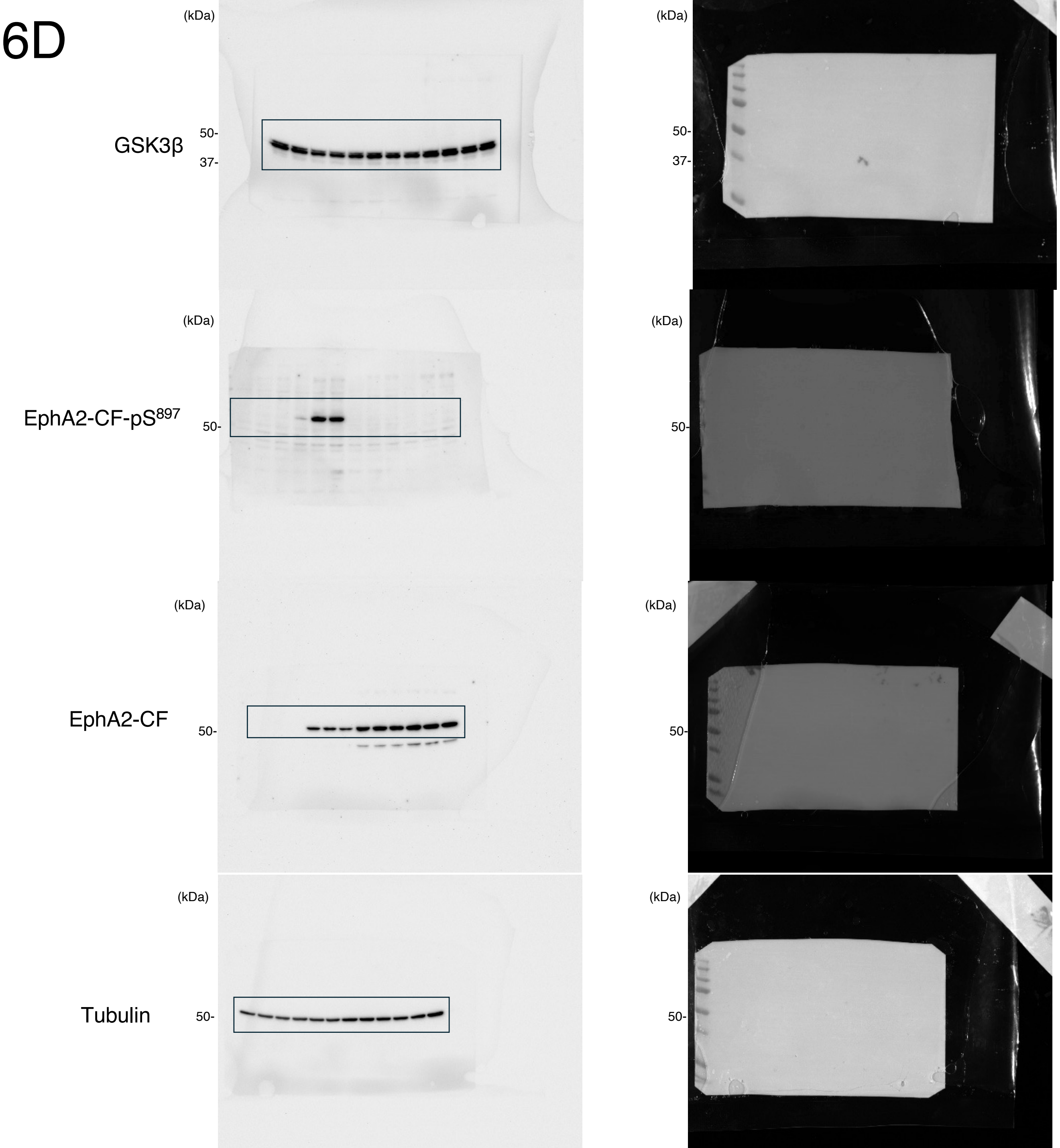
(kDa)
GSK3β
50-
37-
(kDa)
50-
37-
(kDa)
EphA2-CF-pS897
50-
(kDa)
50-
(kDa)
EphA2-CF
50-
(kDa)
50-
(kDa)
Tubulin
50-
(kDa)
50-

Fig6E

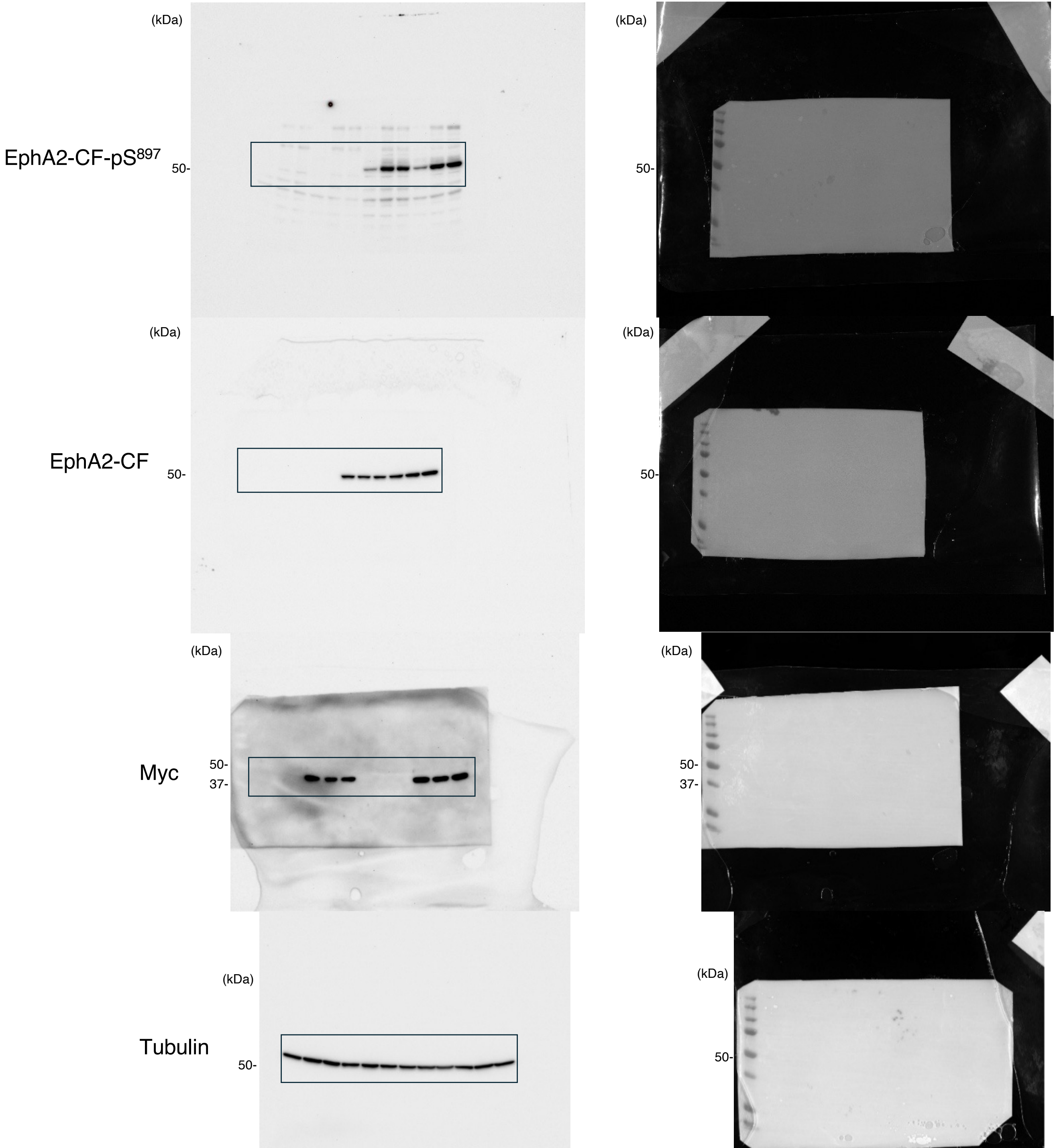

EphA2-CF-pS897
(kDa)
50-
EphA2-CF
(kDa)
50-
Myc
(kDa)
50-
37-
Tubulin
(kDa)
50-

Fig7D

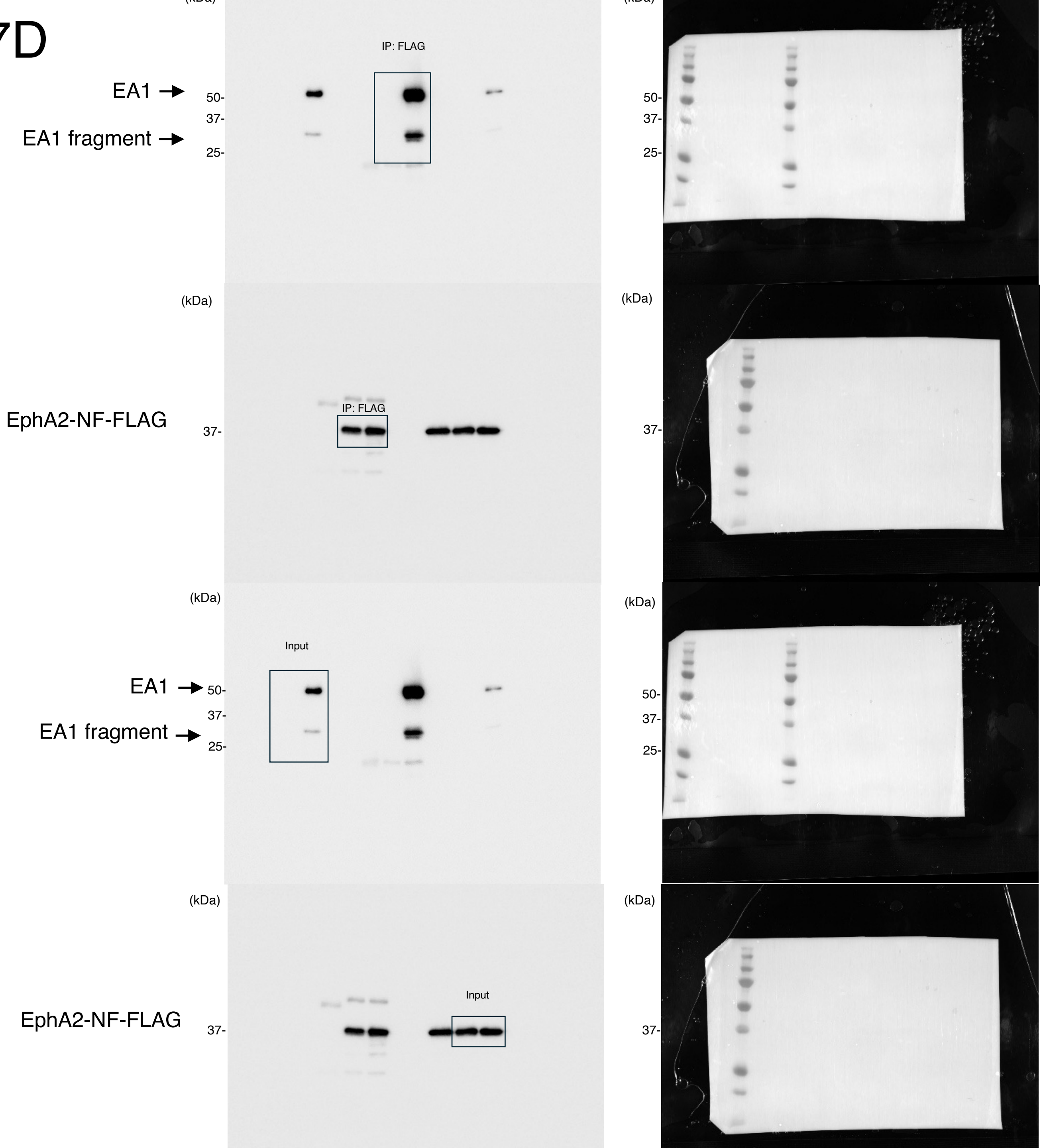
(kDa)
IP: FLAG
EA1
EA1 fragment
50-
37-
25-
(kDa)
IP: FLAG
EphA2-NF-FLAG
37-
(kDa)
Input
EA1
EA1 fragment
50-
37-
25-
(kDa)
Input
EphA2-NF-FLAG
37-

Fig7E

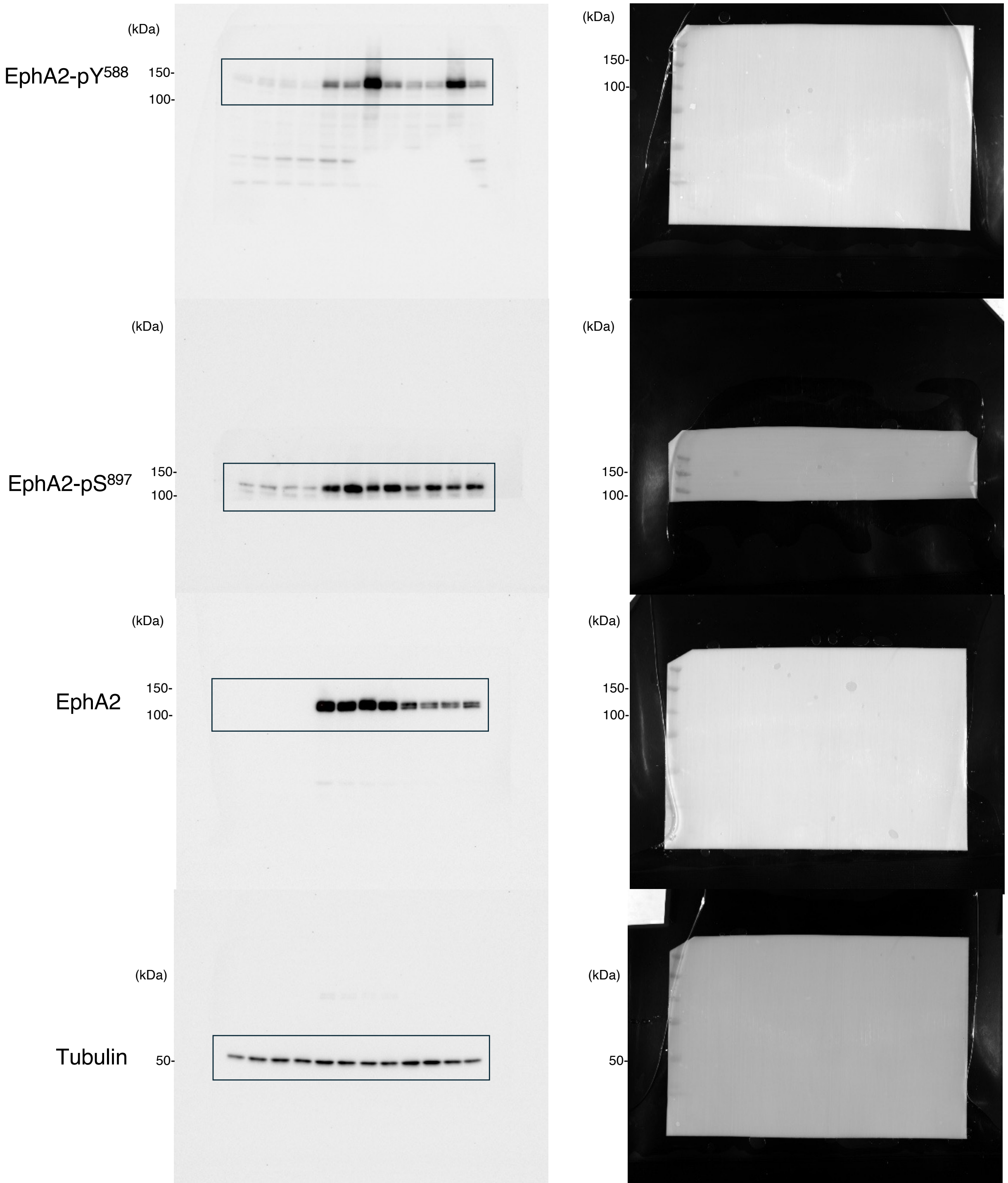
(kDa)
EphA2-pY588
150-
100-
(kDa)
150-
100-
(kDa)
EphA2-pS897
150-
100-
(kDa)
150-
100-
(kDa)
EphA2
150-
100-
(kDa)
150-
100-
(kDa)
Tubulin
50-
(kDa)
50-

FigS1

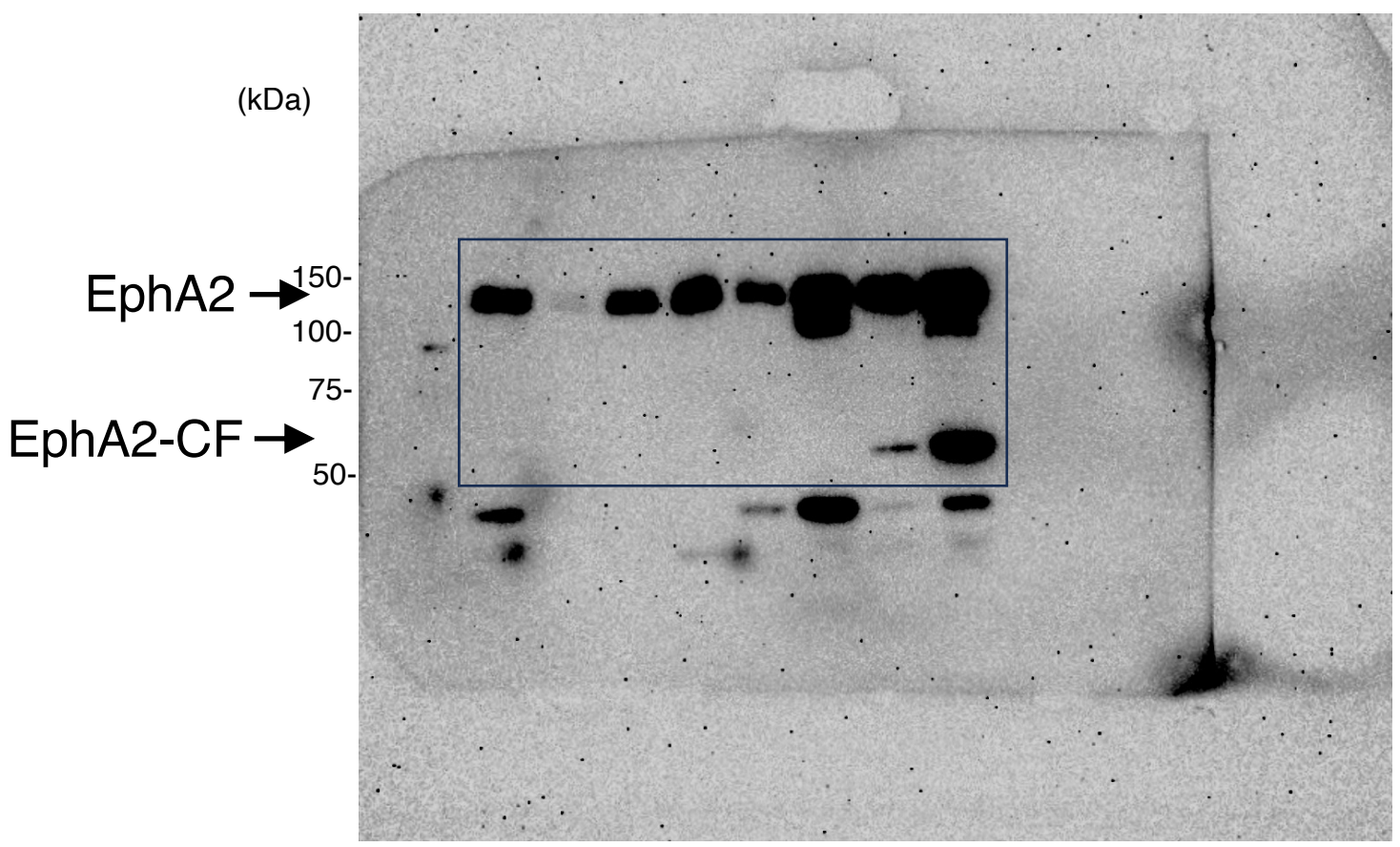

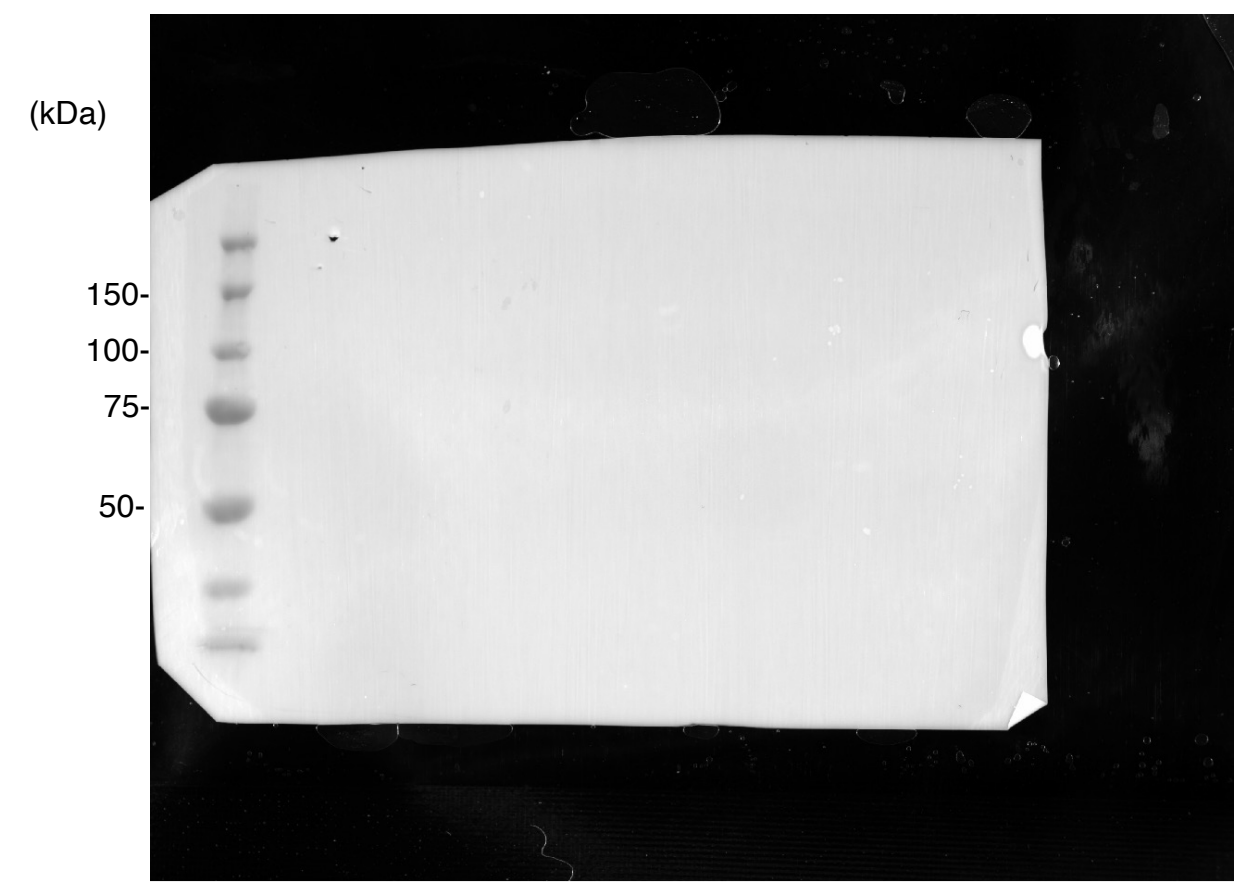

FigS2

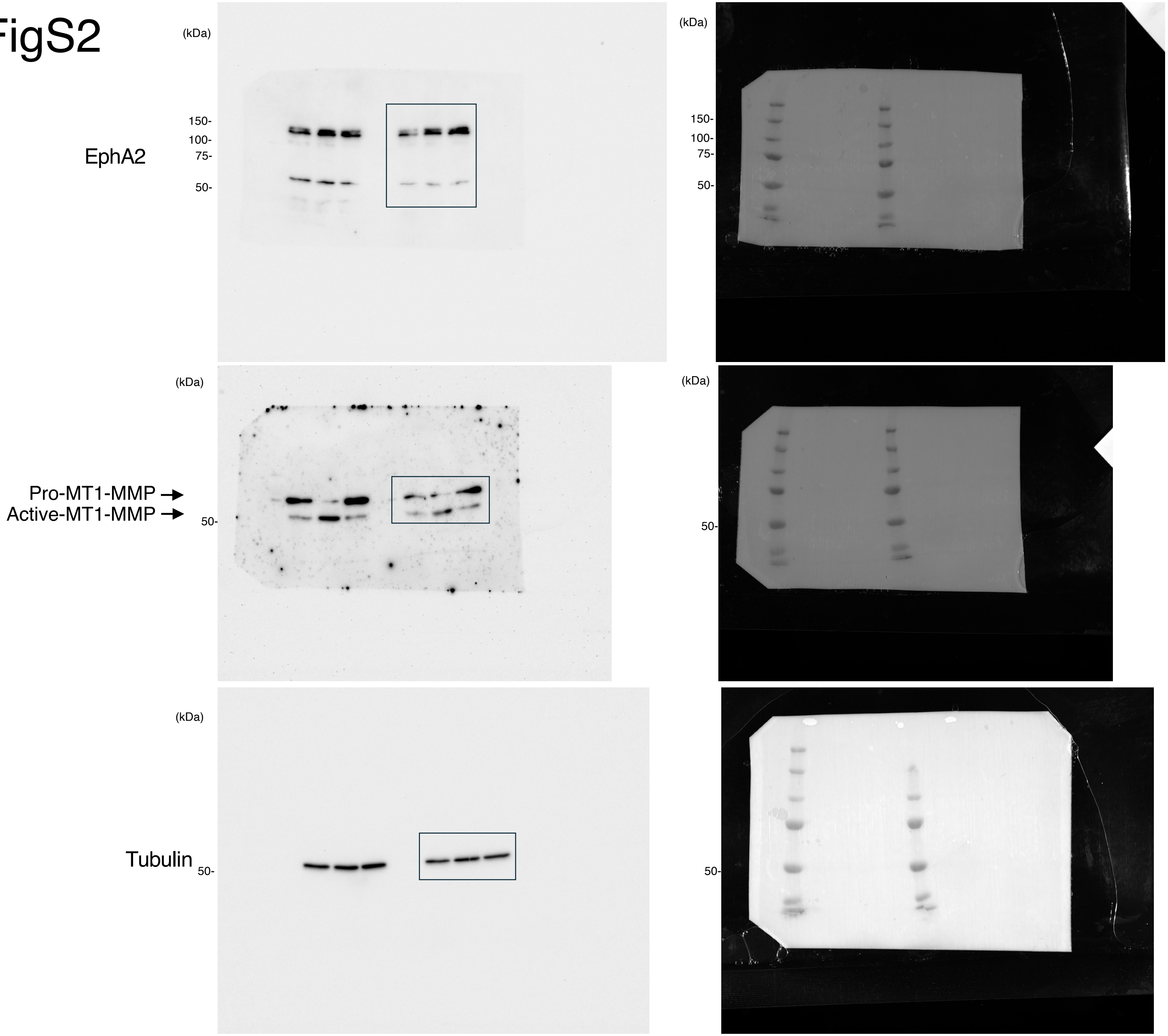

(kDa)
150-
100-
75-
50-
EphA2
(kDa)
150-
100-
75-
50-
(kDa)
Pro-MT1-MMP
Active-MT1-MMP
50-
(kDa)
50-
(kDa)
Tubulin
50-
50-

FigS5 A

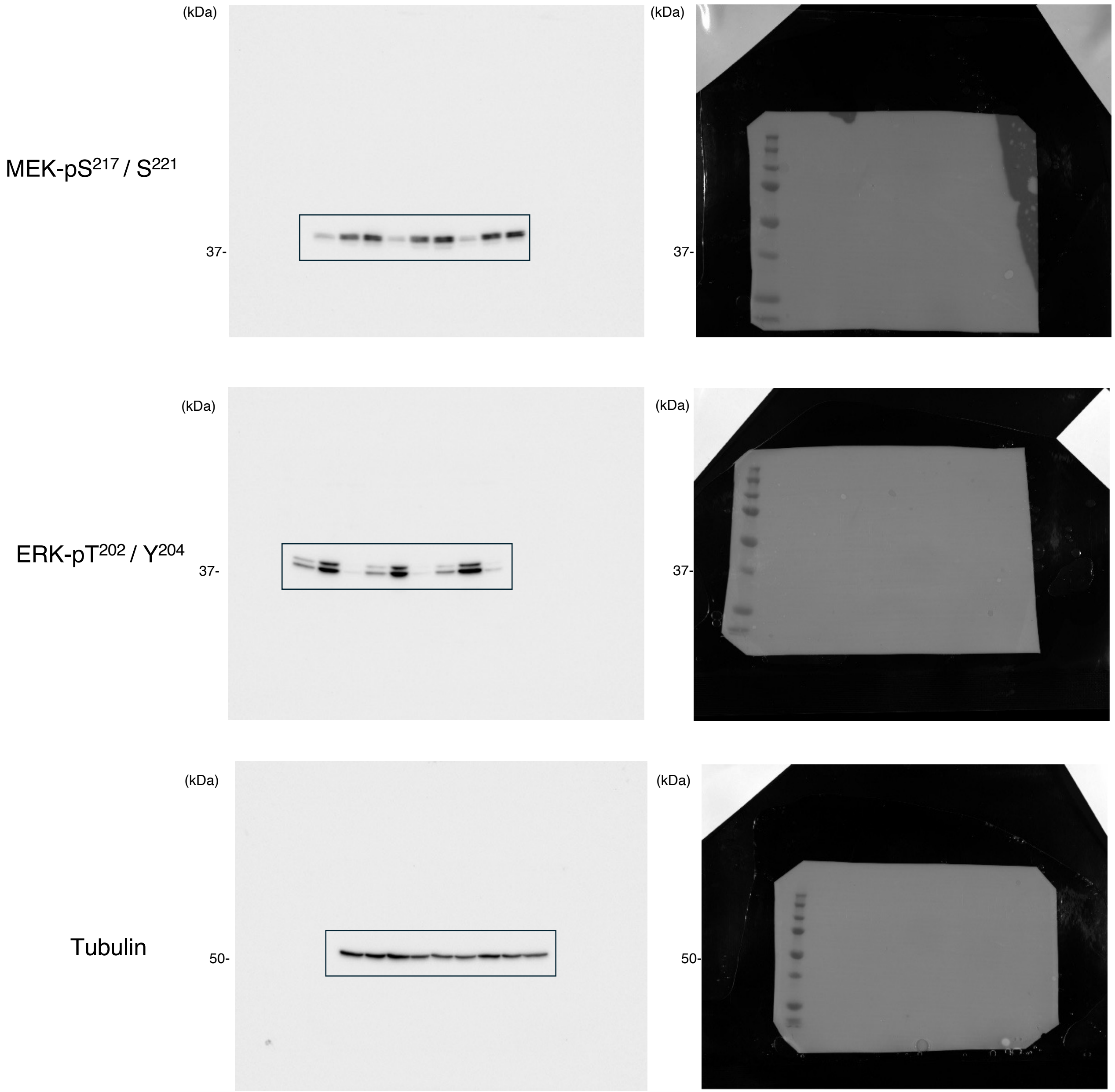

FigS5 B

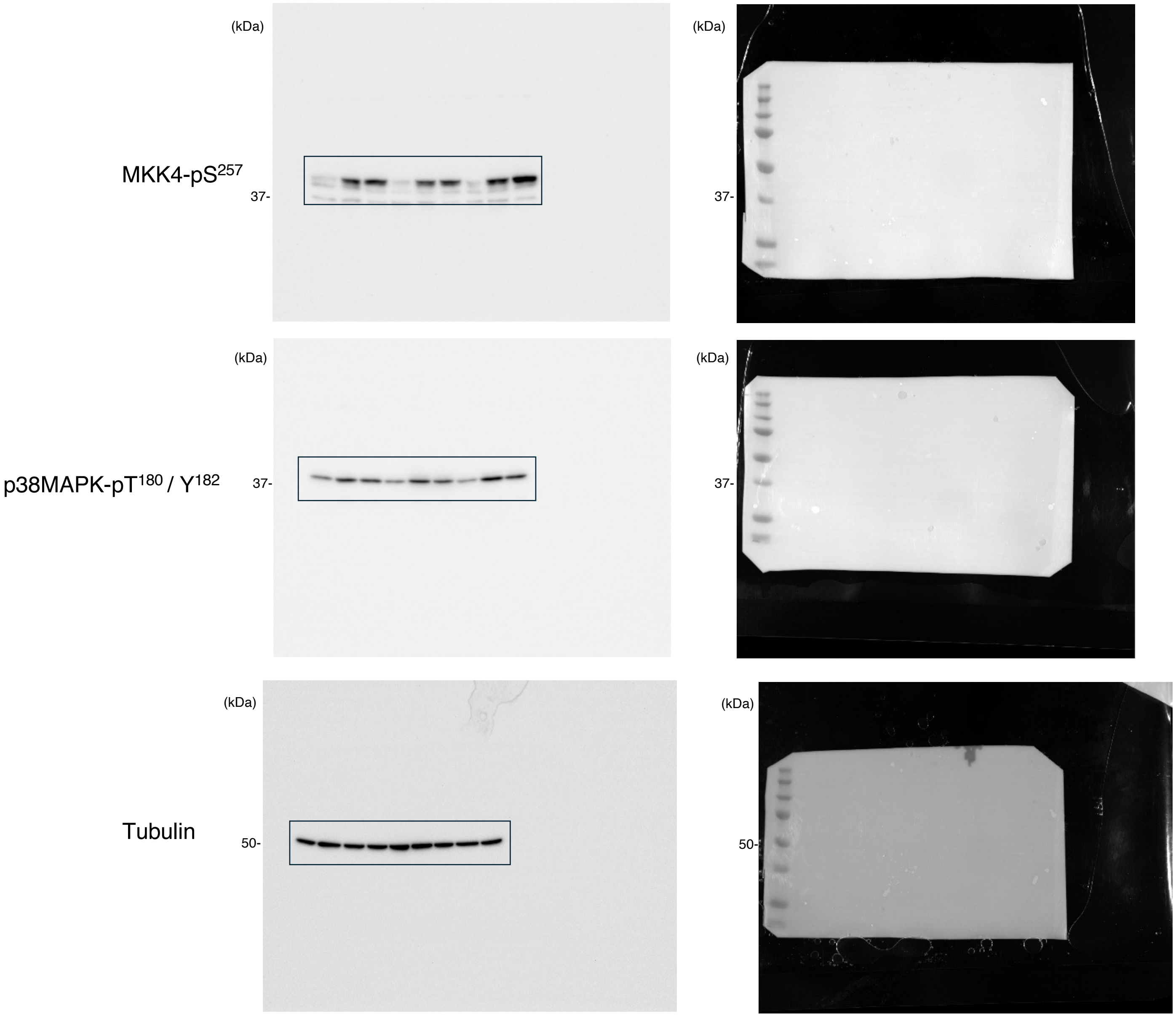
(kDa)
MKK4-pS257
37-
(kDa)
37-
(kDa)
p38MAPK-pT180 / Y182
37-
(kDa)
37-
(kDa)
Tubulin
50-
(kDa)
50-

FigS5 C

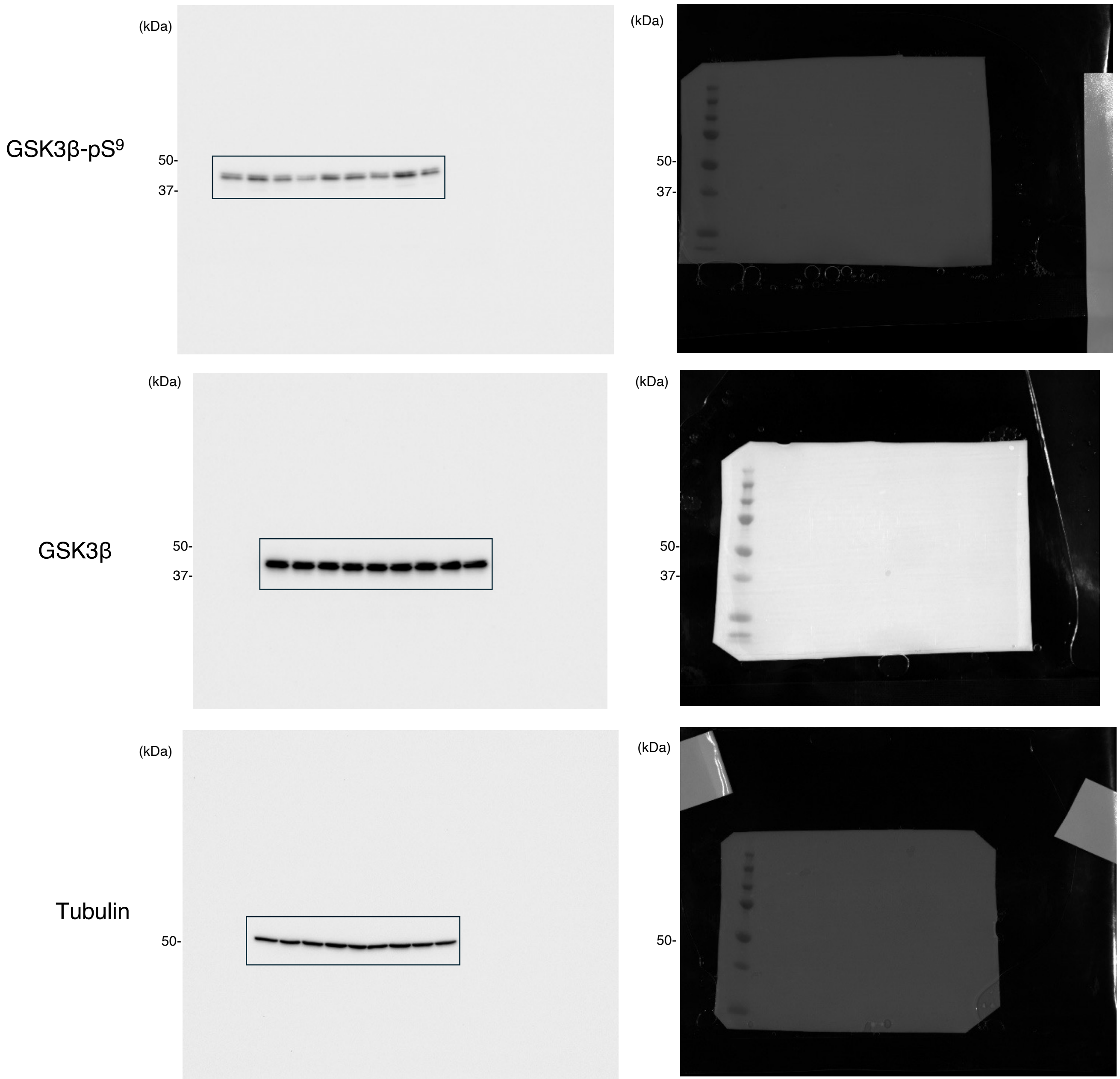
(kDa)
GSK3β-pS9
50-
37-
(kDa)
50-
37-
(kDa)
GSK3β
50-
37-
(kDa)
50-
37-
(kDa)
Tubulin
50-
(kDa)
50-

FigS7 A

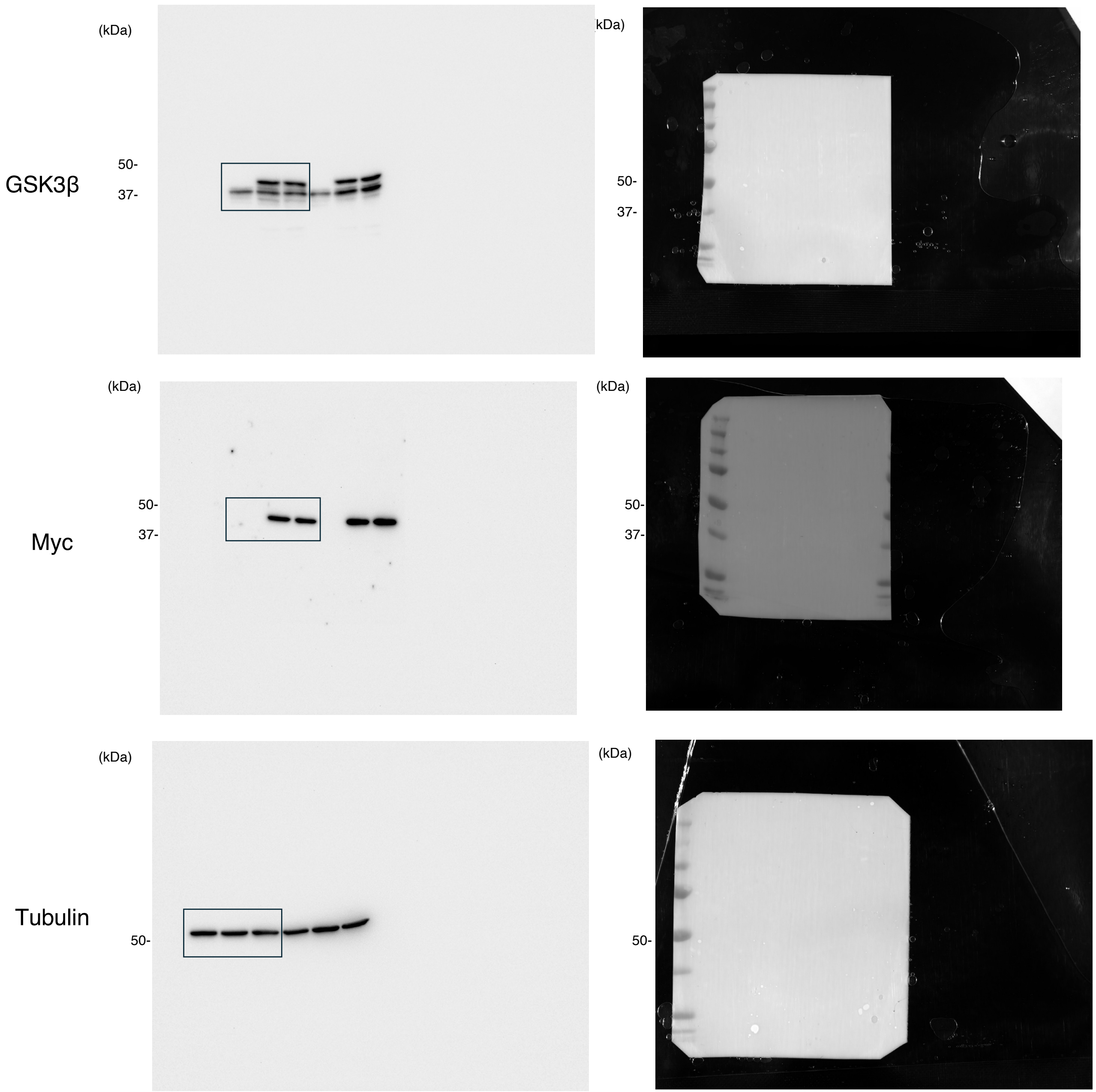

Supplement: Supplementary file 5 [file 41419_2026_9024_MOESM5_ESM.pdf]
